# Zeolite-encaged mononuclear copper centers catalyze CO_2_ selective hydrogenation to methanol

**DOI:** 10.1093/nsr/nwad043

**Published:** 2023-02-20

**Authors:** Yuchao Chai, Bin Qin, Bonan Li, Weili Dai, Guangjun Wu, Naijia Guan, Landong Li

**Affiliations:** Key Laboratory of Advanced Energy Materials Chemistry of Ministry of Education, College of Chemistry, Nankai University, Tianjin 300071, China; Key Laboratory of Advanced Energy Materials Chemistry of Ministry of Education, College of Chemistry, Nankai University, Tianjin 300071, China; CAS Key Laboratory of Science and Technology on Applied Catalysis, Dalian Institute of Chemical Physics, Chinese Academy of Sciences, Dalian 116023, China; School of Materials Science and Engineering, Nankai University, Tianjin 300350, China; Key Laboratory of Advanced Energy Materials Chemistry of Ministry of Education, College of Chemistry, Nankai University, Tianjin 300071, China; School of Materials Science and Engineering, Nankai University, Tianjin 300350, China; Key Laboratory of Advanced Energy Materials Chemistry of Ministry of Education, College of Chemistry, Nankai University, Tianjin 300071, China; School of Materials Science and Engineering, Nankai University, Tianjin 300350, China

**Keywords:** CO_2_ hydrogenation, zeolite, methanol, catalysis, mononuclear copper

## Abstract

The selective hydrogenation of CO_2_ to methanol by renewable hydrogen source represents an attractive route for CO_2_ recycling and is carbon neutral. Stable catalysts with high activity and methanol selectivity are being vigorously pursued, and current debates on the active site and reaction pathway need to be clarified. Here, we report a design of faujasite-encaged mononuclear Cu centers, namely Cu@FAU, for this challenging reaction. Stable methanol space-time-yield (STY) of 12.8 mmol g_cat_^-1^ h^-1^ and methanol selectivity of 89.5% are simultaneously achieved at a relatively low reaction temperature of 513 K, making Cu@FAU a potential methanol synthesis catalyst from CO_2_ hydrogenation. With zeolite-encaged mononuclear Cu centers as the destined active sites, the unique reaction pathway of stepwise CO_2_ hydrogenation over Cu@FAU is illustrated. This work provides a clear example of catalytic reaction with explicit structure-activity relationship and highlights the power of zeolite catalysis in complex chemical transformations.

## Teaser

Zeolite with confined mononuclear Cu ions can efficiently catalyze the selective hydrogenation of CO_2_ to methanol by dihydrogen at relatively low reaction temperatures.

## INTRODUCTION

The increasing amount of atmospheric CO_2_ from anthropogenic emission is becoming a serious concern worldwide, as it can cause significant environmental problems like global warming and increased ocean acidity. Among all technically feasible approaches for reducing and recycling CO_2_, the hydrogenation of CO_2_ to green methanol (CH_3_OH) using preferentially renewable hydrogen sources has drawn great attention [1–5]. The target product CH_3_OH can be directly applied in internal combustion engines and fuel cells or be reserved as a versatile chemical feedstock [6–8]. The CO_2_-to-CH_3_OH transformation is challenging due to the relative chemical inertness of CO_2_ and the difficulty in controlling the side reactions to obtain single product CH_3_OH. Many catalyst systems have been explored for CO_2_ selective hydrogenation, including Cu-based catalysts [9–13], noble metal catalysts [14,15] and metal oxide catalysts [16,17], etc. [18–20]. Hence, Cu-based catalysts with unparalleled advantages of low-cost and high-abundance have been intensively investigated in the past decades. Cu/ZnO/Al_2_O_3_ is currently recognized as a benchmark catalyst for CH_3_OH synthesis from the hydrogenation of CO_2_, CO or CO/CO_2_ mixture. The inherent instability and complexity of Cu component as well as the further modifications by promoters like ZnO bring about significant debates on the active Cu sites [21–28] and the reaction pathway thereof [4,29]. Cu-based catalysts with diverse Cu sites generally suffer from low CH_3_OH selectivity owing to the exacerbated reverse water gas shift (RWGS) reaction to produce CO and the excessive hydrogenation to methane (CH_4_) [30,31]. In practical recycling reactors for CO_2_ hydrogenation, elevated temperatures are employed to obtain reasonable CO_2_ conversion and CH_3_OH space-time-yield (STY), resulting in high energy consumption and further decline in CH_3_OH selectivity. On the other hand, the reaction of CO_2_-to-CH_3_OH is limited by thermodynamic equilibrium, and low temperatures are beneficial to attain both high equilibrium CO_2_ conversion and high CH_3_OH selectivity. The design of highly active Cu-based catalysts for CO_2_ selective hydrogenation to CH_3_OH at low reaction temperatures is therefore possible and urgently needed.

Zeolites are widely employed industrial catalysts and support materials with unique confinement effect [32,33] and ionic environment [34]. Transition metal ions (TMIs) can be accommodated within a zeolite matrix, balancing the negative charges of [AlO_4_]^−^ units, to create more functionalities [35–38]. Interestingly, isolated TMIs can be easily introduced to, and efficiently stabilized by, a zeolite matrix like faujasite *via* a ligand-protected *in situ* hydrothermal route [39,40], providing an opportunity to construct TMIs-containing zeolites toward selective catalysis. Herein, we demonstrate the design of uniform Cu ions confined in faujasite, namely Cu@FAU, for the selective hydrogenation of CO_2_ to CH_3_OH. Cu@FAU catalyst with exclusive mononuclear Cu centers exhibits high CH_3_OH selectivity and STY as well as perfect stability in CO_2_ reduction at relatively low reaction temperatures, fulfilling the basic requirements for industrial applications. With well-defined structure of Cu@FAU model catalyst, the current debates on active Cu sites can be addressed and a clear roadmap of stepwise CO_2_ reduction to CH_3_OH is illustrated.

## RESULTS AND DISCUSSION

### Construction and characterization of Cu(II) confined in zeolite

An *in situ* hydrothermal route was developed to encapsulate Cu complexes in the matrix of faujasite (See Supplementary Information for details) and Cu ions confined in faujasite could be obtained *via* the calcination removal of organic ligands. Post-synthesis modulation was performed and trace exchangeable Cu ions were selectively removed through repeated ion exchange with NaNO_3_ aqueous solution, leaving stable Cu ions confined in faujasite. X-ray diffraction (XRD) patterns identify the typical FAU topology in pure-phase (Fig. S1) and Ar sorption isotherms reveal the uniform microporous structure (Fig. S2) of Cu@FAU. Microscopy analyses indicate the characteristic octahedral faujasite morphology with crystal size of 1–2 μm and the ultra-dispersion of Cu species with loading of ∼4.5 wt% (Figs S3–S5, Table S1).

**Figure 1.**
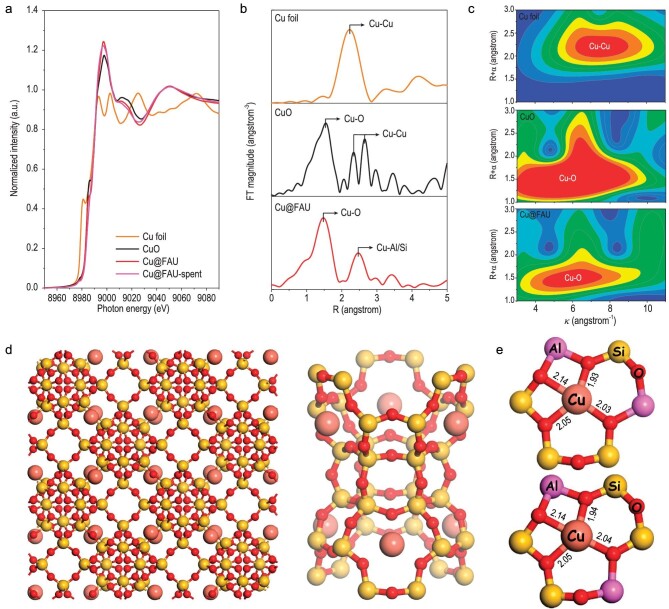
Fine structure of Cu@FAU model catalyst. (a) Cu K-edge XANES spectra of Cu foil, CuO, Cu@FAU and spent Cu@FAU. (b) FT k^2^-weighted EXAFS spectra of Cu foil, CuO and Cu@FAU. (c) WT EXAFS oscillations of Cu foil, CuO and Cu@FAU. (d) Schematic view of Cu@FAU from synchrotron XRD Rietveld refinement. (e) Local coordination environment of Cu sites in faujasite from DFT calculations with bond length shown in angstroms.

A single Cu^2+^→Cu^+^ reduction peak centered at ∼443 K was observed for Cu@FAU (Figs S6 and S7), while complex Cu^+^→Cu^0^ reduction peaks in the temperature region of 523–873 K were observed for Cu-FAU and Cu/FAU samples [41–43]. That is, uniform Cu ions were formed and efficiently stabilized at specific positions in the zeolite matrix in Cu@FAU, in great contrast to the cases of Cu-FAU and Cu/FAU where significant amounts of CuOx species were present as observed in the TEM images (Fig. S8). The valance state of +2 in Cu@FAU was confirmed by Cu K-edge X-ray absorption near-edge structure (XANES) spectrum (Fig. 1a) and a prominent peak at ∼2.0 Å due to the first shell of Cu-O unit with average coordination number of 3.8 was obtained in the Fourier-transformed (FT) *k*^2^-weighted extended X-ray absorption fine structure (EXAFS) spectrum (Fig. 1b, Fig. S9 and Table S2) and the wavelet-transformed (WT) EXAFS oscillations (Fig. 1c). EPR spectrum (Fig. S10) shows a signal of a high-field peak centered at ∼3380 G with EPR parameters g_‖_ = 2.35 (g_‖_ = 2.05) and A = 176 G, assignable to Cu^2+^ species residing in, or close to, the six-membered rings with two aluminum T-sites [44]. The observed signal should be broad and unresolved due to the magnetic dipole interactions from individual Cu ions in close proximity or Cu particles, while the latter possibility could be excluded by microscopic observations (Fig. S5). The fine structure of Cu@FAU and the exact location of Cu sites in faujasite were identified by synchrotron XRD (Fig. S11). According to the results from Rietveld refinement, the Cu sites exclusively situated in the center of the six-membered ring would be shared by the sodalite cage and supercage of zeolite (Fig. 1d). The possible structure of Cu^2+^ in faujasite was also screened by density functional theory (DFT) calculations and the configuration of Cu^2+^ sitting in the six-membered rings containing Al pairs in the *para*- or *meta-*positions could be optimized (Fig. S12). The local coordination environment of Cu sites in faujasite from DFT calculations is shown in Fig. 1e, and the bonding parameters are in good consistency with those from synchrotron X-ray absorbance spectroscopy (Table S2). These results demonstrate the successful construction of Cu@FAU containing uniform and well-defined mononuclear Cu sites, which is analogous to a typical coordination compound with Cu^2+^ as the central ion and faujasite framework as the ligand.

### CO_2_ selective hydrogenation to methanol over Cu@FAU

**Figure 2.**
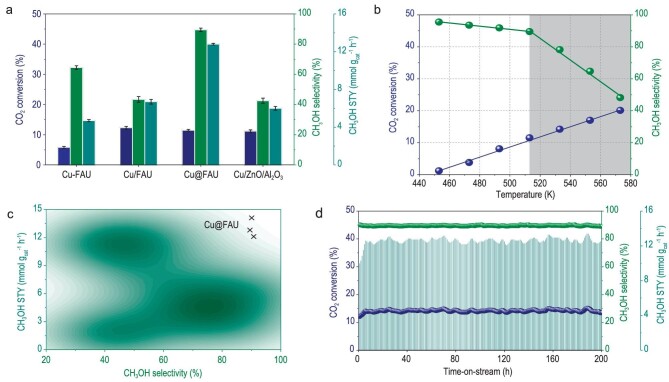
Catalytic performance of Cu@FAU in CO_2_ selective hydrogenation. (a) Representative Cu-based catalysts in CO_2_ hydrogenation. Reaction conditions: 0.15 g catalyst, H_2_/CO_2_ = 3/1, 3 MPa, 513 K, GHSV = 12 000 h^−1^. (b) Temperature-dependent behaviors of Cu@FAU catalyst in CO_2_ hydrogenation. Reaction conditions: 0.15 g catalyst, H_2_/CO_2_ = 3/1, 3 MPa, GHSV = 12 000 h^−1^. (c) Literature survey of Cu-based catalysts for CO_2_ hydrogenation. CH_3_OH selectivity and STY plotted for comparison. (d) Stability test of Cu@FAU catalyst in CO_2_ hydrogenation. Reaction conditions: 0.15 g catalyst, H_2_/CO_2_ = 3/1, 3 MPa, 513 K, GHSV = 12 000 h^−1^.

Figure 2a shows the results of CO_2_ hydrogenation over representative Cu-based catalysts under relatively mild reaction conditions, i.e. at 513 K and in the feed gas of 3.0 MPa CO_2_-H_2_ (H_2_/CO_2_ = 3 : 1). All Cu-containing zeolites, namely Cu-FAU, Cu/FAU and Cu@FAU, can catalyze the CO_2_-to-CH_3_OH transformation, with CO and methane as major byproducts from RWGS and methanation reactions (Fig. S13), respectively. Cu@FAU is the most active catalyst with 11.5% CO_2_ conversion and 89.5% CH_3_OH selectivity, offering a CH_3_OH STY of 12.8 mmol g_cat_^-1^ h^-1^ distinctly higher than that of Cu-FAU (4.7 mmol g_cat_^-1^ h^-1^) and Cu/FAU (6.7 mmol g_cat_^-1^ h^-1^). Due to the absence of Brønsted acid sites (Figs S14 and S15) and the relatively low reaction temperature employed, the formation of dimethyl ether from methanol dehydration can be greatly suppressed, as confirmed by methanol feeding experiment (Fig. S16). The catalytic performance of Cu-containing zeolites seems to be controlled by specific Cu sites and their chemical environment. Notably, similar CO_2_ conversion was achieved with Cu@FAU (4.5wt% Cu) and commercial Cu/ZnO/Al_2_O_3_ (63.0wt% Cu) catalysts despite the huge difference in Cu loading. Cu@FAU exhibits significantly higher CH_3_OH selectivity than Cu/ZnO/Al_2_O_3_ at 473–553 K with comparable CO_2_ conversions (Fig. S17). Cu@FAU surpasses commercial Cu/ZnO/Al_2_O_3_ catalyst in CO_2_ selective hydrogenation at low reaction temperatures.

The temperature-dependent behaviors of Cu@FAU catalyst in CO_2_ hydrogenation are shown in Fig. 2b. The CO_2_ conversion increases almost linearly with increasing reaction temperature from 453 to 573 K, and, meanwhile, two-stage declines in CH_3_OH selectivity are observed, namely the mild declines from 453 to 513 K and the sharp declines from 513 to 573 K. The reaction temperature of 513 K can be optimized in view of both CO_2_ conversion and CH_3_OH selectivity. The second stage decline in the CH_3_OH selectivity should be related to the reduction of Cu^2+^ to Cu^+^, as revealed by *in situ* near-ambient pressure X-ray photoelectron spectroscopy (Fig. S18). Higher pressure and gas hourly space velocity (GHSV) are beneficial to the methanol selectivity (Figs S19 and S20), and CH_3_OH selectivity can be promoted to 92.5% with optimized reaction parameters. The catalytic performance of Cu@FAU, in terms of CH_3_OH selectivity and STY, is superior to all known Cu-based catalysts under comparable reaction conditions (Fig. 2c, Table S3), and, more importantly, the remarkable catalytic performance is achieved with Cu as a single active component free of modifiers like zinc. Cu@FAU catalyst demonstrates good stability and no activity loss or selectivity decline can be observed for over 200-h run of CO_2_ hydrogenation (Fig. 2d, carbon balance >95%), in significant contrast to Cu-FAU (Fig. S21) and Cu/FAU (Fig. S22). Stability is a fatal issue for CH_3_OH synthesis from CO and/or CO_2_ hydrogenation, and Cu-based catalysts generally suffer from rapid deactivation due to metal sintering. For Cu@FAU catalyst, the isolated Cu ions are efficiently stabilized by zeolite matrix and their coordination environment can be well preserved in long-term running, as confirmed by EXAFS analyses (Table S2, Fig. S23). Overall, Cu@FAU appears to be a qualified catalyst for CH_3_OH production from CO_2_ hydrogenation at low reaction temperatures, offering high CH_3_OH selectivity and STY as well as perfect stability.

### Mechanistic insights into CO_2_ selective hydrogenation to methanol

**Figure 3.**
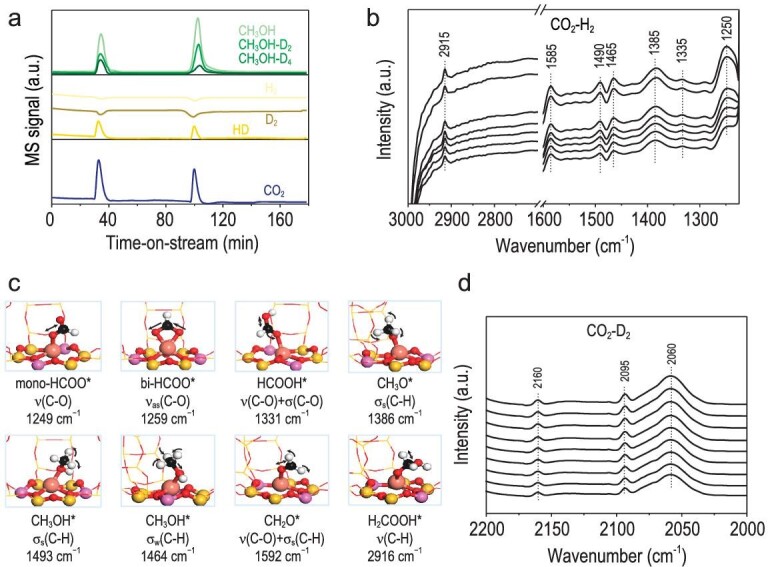
Characteristics of CO_2_ selective hydrogenation over Cu@FAU catalyst. (a) Mass spectrometry responses of CO_2_ pulses fed to Cu@FAU in H_2_-D_2_ stream. Reaction conditions: 0.2 g catalyst, 0.4 MPa, 513 K, 5 mL/min CO_2_, 15 mL/min H_2_-D_2_ (1/1). (b) *In situ* DRIFT spectra of surface species formed on Cu@FAU in CO_2_-H_2_ stream. Time-dependent spectra recorded within 30 min, from light to dark curves. Reaction conditions: 0.02 g catalyst, 3.0 MPa, 513 K, 5 mL/min CO_2_, 15 mL/min H_2_. (c) Calculated structure of key surface intermediates and their vibration frequencies. (d) *In situ* DRIFT spectra of surface species formed on Cu@FAU in CO_2_-D_2_ stream. Reaction conditions: 0.02 g catalyst, 3.0 MPa, 513 K, 5 mL/min CO_2_, 15 mL/min D_2_.

**Figure 4.**
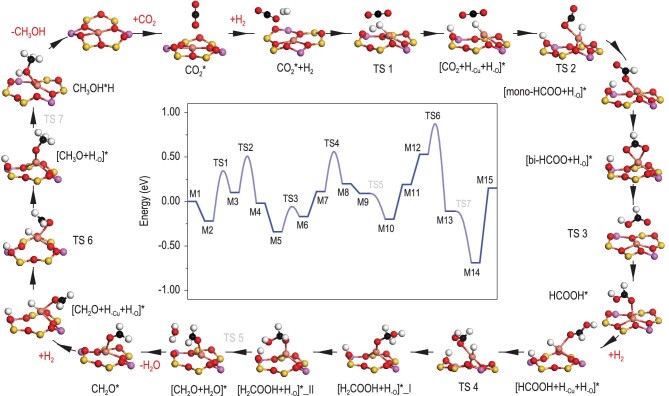
Reaction pathway of CO_2_ hydrogenation to CH_3_OH on mononuclear Cu centers. Elementary reaction steps of CO_2_ hydrogenation over Cu@FAU model catalyst and calculated Gibbs free energy profile with ZPE correction at 0 K.

CO_2_ hydrogenation generally requires both CO_2_ and H_2_ activation, followed by the stabilization of reaction intermediates for controllable hydrogenation. Dihydrogen cannot be activated on Cu@FAU at the reaction temperature of 513 K, as indicated by the absence of HD signal (m/z = 3) in the H_2_-D_2_ stream (Fig. 4a) [45]. Upon the introduction of CO_2_ pulses, the signals of H_2_ and D_2_ decline while the HD signal appears, accompanied by the formation of CH_3_OH and deuterated CH_3_OH (control experiment shown in Figs S24 and S25). These observations clearly demonstrate the CO_2_-assisted dihydrogen activation on Cu@FAU and the subsequent hydrogenation of CO_2_ to CH_3_OH. The surface species involved in the hydrogenation process were then monitored by *in situ* diffuse reflectance infrared Fourier transform spectroscopy (DRIFTS). A series of organic surface species were observed and most of these species reached dynamic equilibrium at the early stage of reaction (Fig. 3b). Typically, the infrared band at 1250 cm^−1^ is assigned to the C–O asymmetric stretching vibrations of mono- or bidentate HCOO^*^ species, and the band at 1335 cm^−1^ assigned to the C–O asymmetric stretching vibrations of HCOOH^*^ species with contribution from bending vibrations. The band at 1385 cm^−1^ is due to the bending vibrations of CH_3_O^*^ species and the paired bands at 1465 and 1495 cm^−1^ due to the bending vibrations of CH_3_OH^*^ species. The band at 1385 cm^−1^ is related to the C–O asymmetric stretching vibrations of CH_2_O^*^ with adjacent co-adsorbed H_2_O and the contribution from C–H bending vibrations. The band at 2915 cm^−1^ is explicitly related to the C–H stretching vibrations of H_2_COOH^*^ species [17,19,46,47]. DFT calculations were employed to support the above assignments, and the structure of key organic species and their vibration frequencies are summarized in Fig. 3c and Table S4. In deuterium labeling experiments, the C-D stretching vibrations of DCOO^*^ (2160 cm^−1^), DCOOD^*^ (2095 cm^−1^) and CD_3_O^*^ (2060 cm^−1^) species were observed (Fig. 3d) [17,48]. All these observations hint to the stepwise CO_2_ reduction by dihydrogen on the Cu@FAU, which appears to be quite different to the conventional CO pathway [24]. Accordingly, Cu@FAU catalyst shows very low activity in the hydrogenation of CO, with ∼2.5% CO conversion at 513 K (Fig. S26).

DFT calculations were finally employed to clarify the reaction pathway of CO_2_ hydrogenation to CH_3_OH over Cu@FAU, with mononuclear Cu centers confined in faujasite as the catalytically active sites. In the first step, gaseous CO_2_ adsorbs on the Cu site in a linear configuration (ln-CO_2_^*^) with adsorption energy of −0.22 eV at 0 K, followed by the adsorption of dihydrogen. The dihydrogen undergoes facile dissociation into a hydride bonded to the Cu site and a proton bonded to adjacent O site *via* TS1 (*E*_a_ = 0.75 eV, *E*_r_ = 0.32 eV). Clearly, the dihydrogen is activated by classical Lewis pairs (Cu–O) with the assistance from adsorbed CO_2_ species, as confirmed by the pulse-response experiments in Fig. 3a. The hydride transfer to the C atom of the ln-CO_2_^*^ results in the formation of the monodentate formate (mono-HCOO^*^) *via* TS2 (*E*_a_ = 0.64 eV, *E*_r_ = −0.12 eV). The mono-HCOO^*^ rapidly transforms into the bidentate formate (bi-HCOO^*^, *E*_r_ = −0.29 eV), which is further protonated to the HCOOH^*^via TS3 (*E*_a_ = 0.37 eV, *E*_r_ = 0.17 eV). The second dihydrogen molecule then dissociates into a hydride and a proton at the Cu–O pair site (assisted by adsorbed HCOOH) and the HCOOH^*^ is hydrogenated to the H_2_COOH^*^via TS4 (*E*_a_ = 0.65 eV, *E*_r_ = 0.09 eV). Through a simple rotation, the H_2_COOH^*^ reacts with the proton to form the CH_2_O^*^ and the H_2_O^*^via TS5 (*E*_a_ = 0.00 eV, *E*_r_ = −0.29 eV). The H_2_O^*^ leaves the active site with the desorption energy of 0.39 eV and enables the third dihydrogen dissociation at the Cu-O pair site (assisted by adsorbed CH_2_O). The CH_2_O^*^ is hydrogenated to the CH_3_O^*^via TS6 (*E*_a_ = 0.63 eV, *E*_r_ = −0.64 eV) and the CH_3_OH^*^ is formed by the protonation of the CH_3_O^*^via TS7 (*E*_a_ = 0.00 eV, *E*_r_ = −0.58 eV). Finally, the CH_3_OH^*^ desorbs from the active site (*E*_r_ = 0.84 eV) and the catalytic cycle ends. For a direct view, the complete reaction pathway of CO_2_ hydrogenation to CH_3_OH over Cu@FAU model catalyst and the calculated Gibbs free energy profile are shown in Fig. 4 (adsorption energy of key intermediates listed in Table S5 and Table S6). Some of the key reaction intermediates like HCOOH^*^, H_2_COOH^*^, CH_3_O^*^ and CH_3_OH^*^ have been successfully captured by *in situ* DRIFTS, as shown in Fig. 3d.

According to above analyses, all the energy barriers from TS1 to TS7 are less than 0.75 eV and the reaction energies are less than 0.32 eV, indicating that CH_3_OH production from CO_2_ hydrogenation on zeolite confined mononuclear Cu centers is thermodynamically and kinetically favorable. The first CO_2_-assisted dihydrogen dissociation has the highest activation barrier, and it should be the rate-determining step for the overall reaction. At the optimized temperature of 513 K, the energy barriers and reaction energies are also reasonable (Fig. S27). The oxidation state of copper species shows dynamic changes during reaction while the cationic state of copper can be well preserved, as confirmed by the variations of Bader charge (Fig. S28). The high catalytic activity of the Cu@FAU originates from the unique configuration of Cu^δ+^ sitting in the six-membered ring, containing classical Lewis pairs of Cu^δ+^–O^2+^ units. The C-containing intermediates can effectively adsorb on Cu sites and assist the dihydrogen activation on Cu–O pairs for subsequent hydrogenation *via* the so-called associative mechanism [45]. We also search for the bent configuration of CO_2_ (bt-CO_2_^*^) by structure optimization, which is found to be extremely unstable and undergoes spontaneous transformation to ln-CO_2_^*^ on the Cu site. Thereupon, the traditional protonation of bt-CO_2_^*^ to COOH^*^ followed by dissociation to CO^*^ [29,49] or bt-CO_2_^*^ direct dissociation to CO^*^ [50] will not occur on Cu@FAU. With well-defined uniform mononuclear Cu cations confined in faujasite, Cu@FAU is completely different to traditional Cu-based catalysts containing a complicated constitution of Cu species and always modified by Zn species [21–28]. For Cu@FAU, the step-wise CO_2_ hydrogenation to CH_3_OH is achieved on zeolite-confined Cu–O Lewis pairs and dihydrogen activation is assisted by adsorbates on Cu sites like CO_2_, HCOOH and CH_2_O. Such unique homogeneous-like mechanism derives unprecedented catalytic performance in CO_2_-to-CH_3_OH transformation at relatively low temperatures (Fig. 2).

## CONCLUSIONS

The selective hydrogenation of CO_2_ to CH_3_OH provides a technically feasible route for CO_2_ recycling and is carbon neutral. The reaction process is very complex and requires the well-balanced C–O dissociation and dihydrogen activation. A complicated catalyst system of Cu/ZnO/Al_2_O_3_ is currently employed to achieve high activity and moderate selectivity to CH_3_OH as well as good catalyst stability, which also brings about significant debates on the active site and reaction mechanism. Herein, we demonstrate that zeolite-encaged uniform mononuclear Cu centers, namely Cu@FAU, can efficiently catalyze the stable CO_2_-to-CH_3_OH transformation at the relatively low reaction temperature of 513 K, offering a high CH_3_OH STY of 12.8 mmol g_cat_^-1^ h^-1^ at selectivity of 89.5%. The reaction sequence of CO_2_ hydrogenation over well-defined Cu@FAU catalyst and the full catalytic cycle are successfully depicted. It is disclosed that all the reaction steps can take place on Cu^δ+^–O^2+^ Lewis pairs confined in zeolites, following the homogeneous-like mechanism. The unique zeolite-confined catalyst system and reaction pathway contribute to the success of CO_2_-to-CH_3_OH process for carbon neutral, and may trigger some new thoughts for other complex chemical transformations.

## MATERIALS AND METHODS

Cu@FAU was synthesized *via* a ligand-protected *in situ* hydrothermal route. The composition of the gel with the molar ratio of 7.8 SiO_2_ : 1 Al_2_O_3_ : 2.2 Na_2_O: 0.6 Cu-TAPTS: 174 H_2_O (TAPTS = 3-[2-(2-aminoethylamino) ethylamino] propyl-trimethoxysilane) was transferred into an autoclave and heated at 373 K for 4 days under static conditions. The solid was collected by centrifugation, washed with water, dried at 353 K overnight and calcined in flowing air at 823 K for 6 h.

XRD patterns of selected zeolite samples were recorded on a Bruker D8 diffractometer. High resolution synchrotron XRD data were collected at Beamline I11 of Diamond Light Source using multi-analysing crystal-detectors and monochromated radiation [λ = 0.826126(2) Å]. TEM images of selected samples were acquired on a FEI Tecnai G2 F20 electron microscope. EPR spectra were collected with a continuous wave X-band Bruker EMX EPR spectrometer. ^1^H magic-angle-spinning nuclear magnetic resonance (MAS NMR) spectra were obtained on a Bruker Avance III spectrometer. *In situ* near ambient pressure XPS were performed on a SPECS NAPXPS spectrometer. XAS spectra were measured at the BL11B, Shanghai Synchrotron Radiation Facility (SSRF).

The catalytic reaction of CO_2_ hydrogenation was carried out in a high-pressure fixed-bed continuous-flow reactor. The products were analyzed using an online gas chromatograph (Shimadzu 2010SE) equipped with a thermal conductivity detector (TCD) and a flame ionization detector (FID). A TDX-01 packed column was connected to the TCD and an RT-Q-BOND-PLOT capillary column was connected to the FID. Product selectivity was calculated on a molar carbon basis, and the TCD and FID signals were correlated by the signal of methane.

## Supplementary Material

nwad043_Supplemental_File
