# Supplementary material for: Zeolite-encaged mononuclear copper centers catalyze CO2 selective hydrogenation to methanol
Source: Natl Sci Rev. 2023 Feb 20;10(7):nwad043. doi: 10.1093/nsr/nwad043 (PMC10401316; doi:10.1093/nsr/nwad043)
Supplement: nwad043_Supplemental_File [file nwad043_supplemental_file.pdf]

Supplementary Information for

Zeolite-encaged mononuclear copper centers catalyze CO_2 selective hydrogenation to methanol

Yuchao Chai,[1#] Bin Qin,[1#] Bonan Li,[3] Weili Dai,[2] Guangjun Wu,[1] Naijia Guan,[2] Landong Li[1,2*]

[1] Key Laboratory of Advanced Energy Materials Chemistry of Ministry of Education, College of Chemistry, Nankai University, Tianjin 300071, P.R. China

[2] School of Materials Science and Engineering, Nankai University, Tianjin 300350, P.R. China

[3] CAS Key Laboratory of Science and Technology on Applied Catalysis, Dalian Institute of Chemical Physics, Chinese Academy of Sciences, Dalian 116023, P.R. China

[*]**Corresponding author.** E-mail: lild@nankai.edu.cn

[#]Equally contributed to this work.

This PDF file includes:

Materials and Methods

Figures S1 to S28

Tables S1 to S5

Supplementary References S1-S39

Materials and Methods

Chemical reagents

Copper nitrate [$Cu(NO_3)_2$, Aladdin], sodium aluminate ($NaAlO_2$, Aladdin), 3-[2-(2-aminoethylamino)ethylamino]propyl-trimethoxysilane (TAPTS, Macklin, CAS: 35141-30-1), sodium hydroxide (NaOH, Aladdin), silica sol (50 wt.% SiO_2, Alfa Aesar), sodium nitrate ($NaNO_3$, Aladdin). All chemical reagents are of analytical grade and used directly without further purification.

Hydrothermal synthesis of Cu@FAU

Cu@FAU was synthesized *via* a ligand-protected *in situ* hydrothermal route. In a typical experiment, 4.13 g $Cu(NO_3)_2$ was added into 98 mL deionized water containing 11.71 g TAPTS and stirred for 30 minutes to obtain the Cu-TAPTS solution. Then, 5.80 g $NaAlO_2$ and 6.05 g NaOH were added into the solution in turn. After stirring for 1 h, 34 g silica sol (50 wt.% SiO_2) was dropwise added into the above mixture under vigorous stirring to form the synthesis gel. Finally, the gel with the molar ratio of 7.8 SiO_2: 1 Al_2O_3: 2.2 Na_2O: 0.6 Cu-TAPTS: 174 H_2O was transferred into an autoclave and heated at 373 K for 4 days under static conditions. The solid was collected by centrifuging, washed with water, dried at 353 K overnight and calcined in flowing air at 823 K for 6 h.

The calcined solid samples were subsequently ion-exchanged with 1 M $NaNO_3$ solution for three times to selectively remove the Cu ions at the exchangeable sites, dried at 353 K overnight, and calcined in flowing air at 823 K for 6 h to derive Cu@FAU sample for catalysis.

Preparation of Cu-FAU and Cu/FAU

Commercial Na-FAU zeolite (Si/Al = 3.5) was employed as zeolite host and Cu species were introduced to the zeolite by ion-exchanged with 1.0 M $Cu(NO_3)_2$ aqueous solution for three times at the constant temperature of 353 K. After each ion-exchange process, the slurry was filtered and washed with distilled water. The final solid product was dried at 353 K overnight and calcined in flowing air at 823 K for 6 h to derive Cu-FAU.

Cu species were also introduced into Na-FAU zeolite (Si/Al = 3.5) by wet impregnation, followed by similar drying and calcination steps. The final product was denoted as Cu/FAU.

Sample characterization

The chemical compositions of samples were analyzed on an IRIS Advantage inductively coupled

plasma atomic emission spectrometer (ICP-AES).

The X-ray diffraction (XRD) patterns of selected zeolite samples were recorded on a Bruker D8 diffractometer using Cu-Kα radiation (λ= 0.1541 nm) in the region of 2θ = 5-50° at a scanning rate of 6 ^{o}/min. High resolution synchrotron X-ray powder diffraction data of selected samples were collected at Beamline I11 of Diamond Light Source using multi-analysing crystal-detectors and monochromated radiation [λ =0.826126(2) Å].

The surface areas of samples were determined by Ar adsorption/desorption isotherms at 87 K collected on a Quantachrome iQ-MP gas adsorption analyser. The total surface area was calculated *via* the Brunauer Emmett Teller (BET) equation and the micropore volume was determined using the t-plot method. Prior to Ar adsorption, the sample of ~0.1 g was desolvated under dynamic vacuum at 473 K for 12 h.

Transmission electron microscopy (TEM) images of selected samples were acquired on a FEI Tecnai G2 F20 electron microscope.

The experiments of temperature-programmed reduction by hydrogen (H_2-TPR) were performed on a Quantachrome ChemBET 3000 chemisorption analyzer. In a typical experiment, the sample of ~0.1 g was calcined in dry air at 823 K for 1 h and cooled to 323 K in flowing Ar. H_2-TPR profile was recorded in flowing 5%H_2/Ar at a heating rate of 10 K /min from 323 to 1123 K.

Electron paramagnetic resonance (EPR) spectra were collected with a continuous wave X-band Bruker EMX EPR spectrometer with the ER 4102ST cavity with a gunn diode microwave source in the field interval 220-400 mT.

The experiments of temperature-programmed desorption of ammonia (NH_3-TPD) were performed on a Quantachrome ChemBet 3000 chemisorption analyzer. In a typical experiment, the sample was saturated with 5% NH_3/He at 323K and then purged with He at the same temperature for 1 h to eliminate the physical absorbed ammonia. The NH_3-TPD profile was recorded in flowing He at a heating rate of 10 K/min from 323 to 873 K.

The solid-state magic-angle-spinning nuclear magnetic resonance (MAS NMR) measurements were performed on a Bruker Avance III spectrometer at resonance frequencies of 400.1 MHz for ^{1}H nuclei. ^{1}H MAS NMR spectra were obtained upon a single-pulse excitation of π/2 with pulse duration of 2.6 μs and a repetition time of 20 s, respectively. The ammonia loading of the dehydrated samples was done on a vacuum line by adsorption of 100 mbar ammonia (Griesinger) at

298 K for 10 min, followed by an evacuation (p < 10^{-2} mbar) at 453 K for 2 h for removing weakly physisorbed ammonia.

In situ near ambient pressure X-ray photoelectron spectra (XPS) of Cu-containing zeolites were performed on a SPECS NAPXPS spectrometer with monochromatic Al Ka X-ray (hν= 1486.6 eV) as the excitation source. The binding energies (± 0.1 eV) were determined with respect to the position of Si 2p peak of SiO_2 at 103.3 eV.

The X-ray absorption spectra (XAS) were measured at the BL11B, Shanghai Synchrotron Radiation Facility (SSRF) (*S1*), including X-ray absorption near-edge structure (XANES) and extended X-ray absorption fine structure (EXAFS) spectra at the Cu K-edge. A Si (111) double-crystal monochromator was used for the energy selection. The energy was calibrated by Cu foil as a reference and all samples were measured in the transmission mode. The Athena software package was used to analyze the data.

Catalytic reaction of CO_2 hydrogenation

The catalytic reaction of CO_2 hydrogenation was carried out in a high-pressure fixed-bed continuous-flow reactor. Typically, catalyst sample of 0.2 g was placed in the quartz reactor, pretreated in Ar at 673 K for 1 h, and cooled down to designated reaction temperature. Afterwards, the reaction was conducted under the reaction conditions of 1.0-4.0 MPa, 453-573 K, $V_{H2}/V_{CO2}/V_{Ar}$ of 72/24/4, and gas hourly space velocity (GHSV) of 8000-20,000 /h. The products were analyzed using an online gas chromatograph (Shimadzu 2010SE) equipped with a thermal conductivity detector (TCD) and a flame ionization detector (FID). A TDX-01 packed column was connected to the TCD and an RT-Q-BOND-PLOT capillary column was connected to the FID. Product selectivity was calculated on a molar carbon basis, and the TCD and FID signals were correlated by the signal of methane.

***In situ* diffuse reflectance infrared Fourier transform spectroscopy**

The reaction of CO_2 hydrogenation to methanol was *in situ* monitored by diffuse reflectance infrared Fourier transform spectroscopy (DRIFTS). The experiments were performed on a Bruker Tensor 27 spectrometer equipped with an *in situ* reaction chamber and a liquid N_2 cooled high sensitivity mercury cadmium telluride detector. Typically, ~20 mg of finely-ground catalyst powders were placed in the reaction chamber and pretreated in Ar at 673 K for 1 h. After cooling down to the designated temperature, the reactant gas mixture containing H_2/CO_2 (3/1) or D_2/CO_2

(3/1) was fed into the chamber at GHSV of 12000 h^{-1}, and time-resolved spectra were recorded at a resolution of 4 cm^{-1} and with an accumulation of 128 scans against blank background.

Computational methods and modeling

The spin-polarized DFT calculations were performed using the Vienna ab initio simulation package (VASP) (*S2*,*S3*). The Perdew-Burke-Ernzerhof (PBE) exchange-correlation functional (*S4*) and the projector-augmented wave (PAW) potentials (*S5*) were used to describe the electron-ion interaction. The Bayesian error estimation functional with van der Waals correlation (BEEF-vdW) (*S6*) and an energy cut-off of 400 eV were employed in this study. All structures were optimized using Γ point. The electronic energy of the supercell was converged to 10-4 eV, and the force on all unconstrained atoms were converged to 0.01 eV Å.

The structure of the Cu@FAU zeolite was built according to the characterization results. All atoms in the structure were allowed to relax. The zero-point energies (ZPE), enthalpies, entropies, and Gibbs free energies were calculated from harmonic frequencies, identical to our previous work (*S7*). Transition states were obtained using the climbing image nudged elastic band (CI-NEB) method (*S8*,*S9*) and confirmed by harmonic frequencies.

Figures & Tables

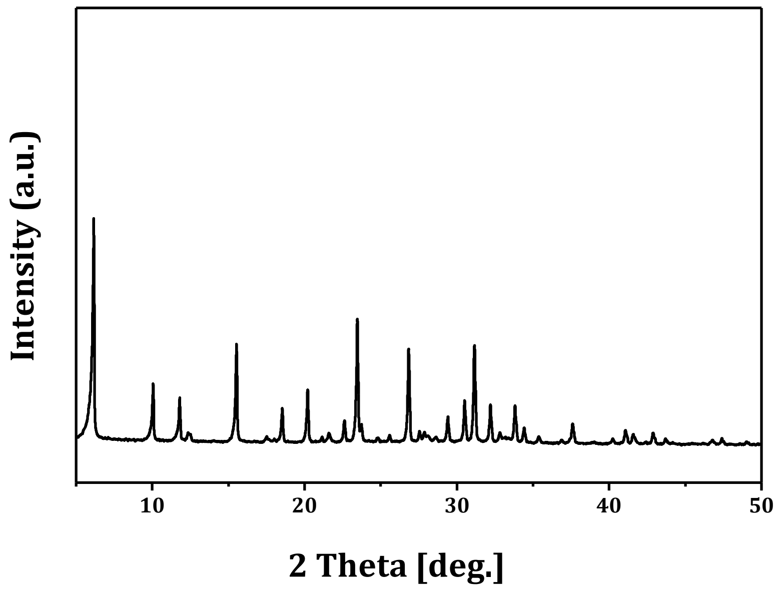

Figure S1 XRD pattern of Cu@FAU zeolite

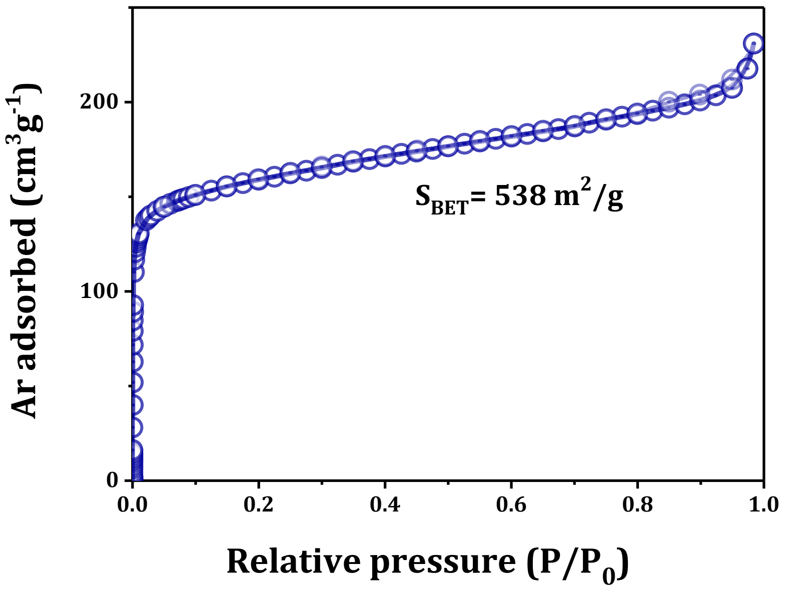

Figure S2 Ar adsorption-desorption isotherms of Cu@FAU at 87 K

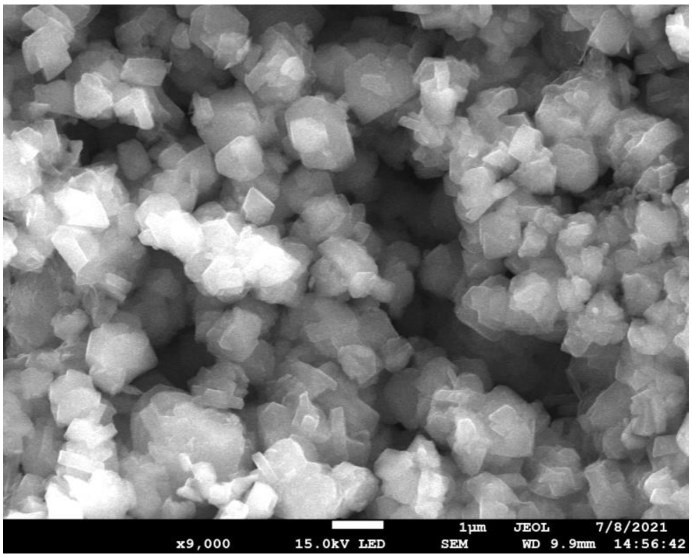

Figure S3 SEM images of Cu@FAU sample

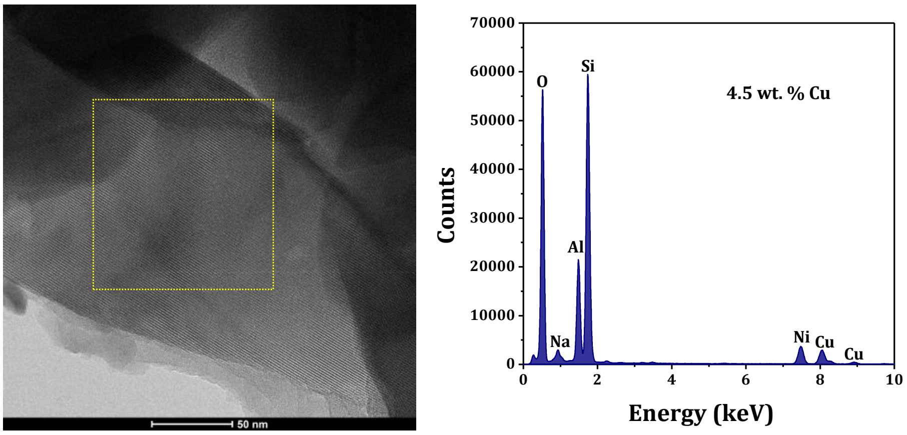

Figure S4 TEM image of Cu@FAU sample with selected area energy dispersive X-ray spectrum

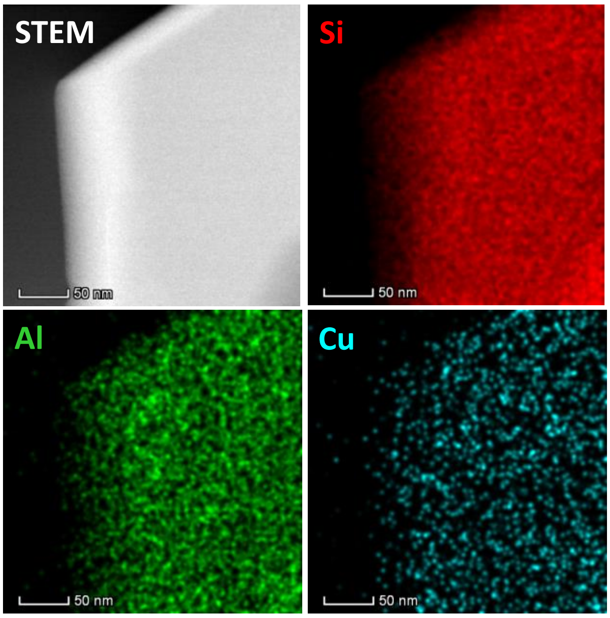

Figure S5 HAADF-STEM image of Cu@FAU and corresponding element mapping

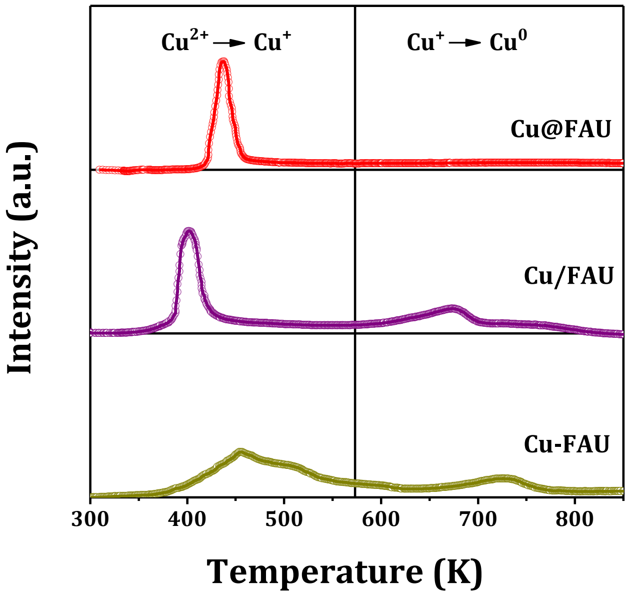

Figure S6 H_2-TPR profiles of Cu-containing FAU zeolites

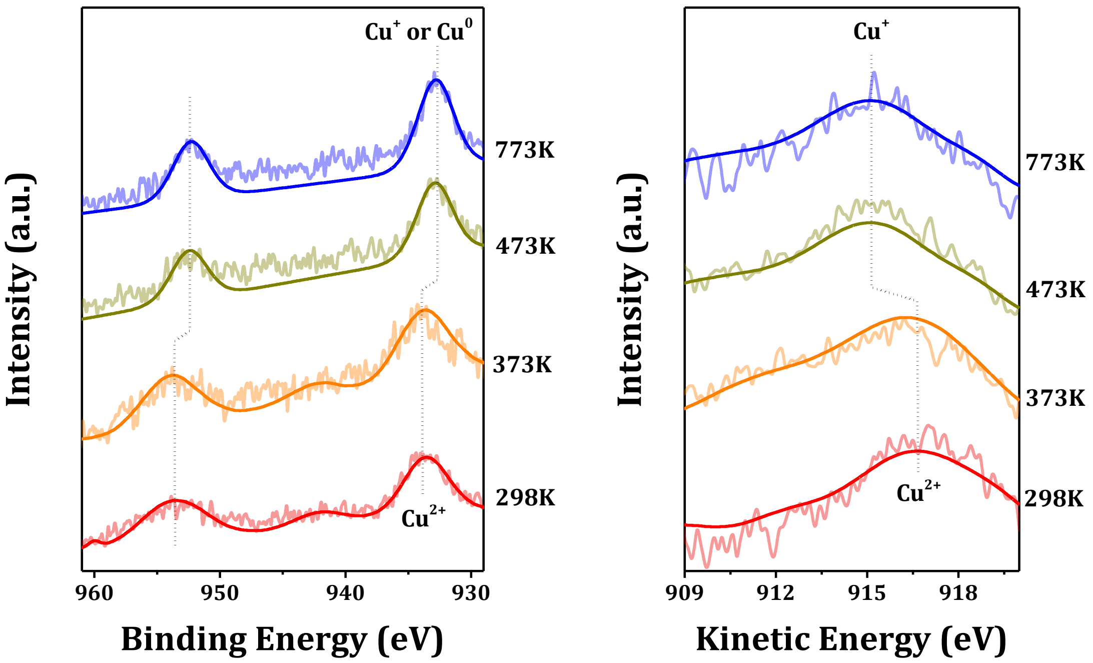

Figure S7 *In situ* near-ambient pressure X-ray photoelectron spectra of Cu@FAU during H_2 reduction

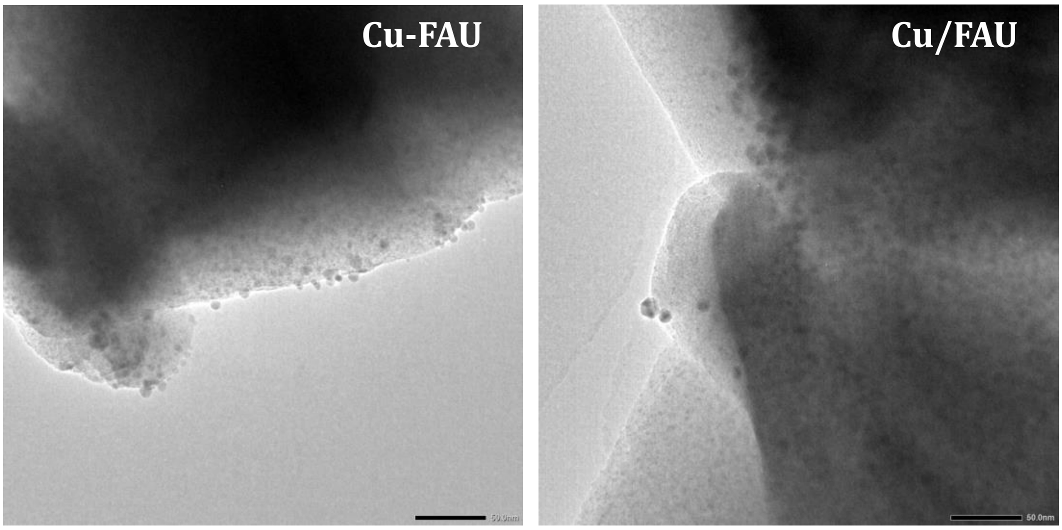

Figure S8 TEM images of Cu-FAU and Cu/FAU samples

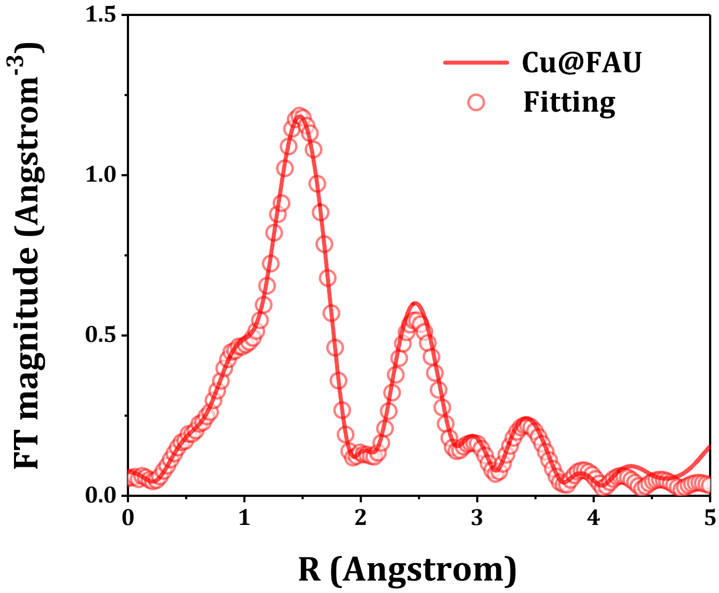

Figure S9 FT EXAFS fitting spectrum of Cu@FAU

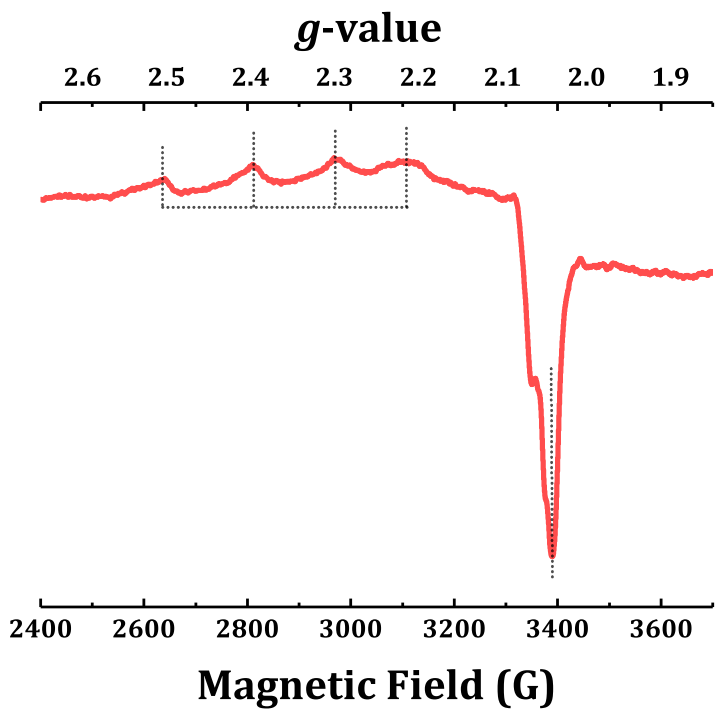

Figure S10 EPR spectrum of Cu@FAU measured at 77 K

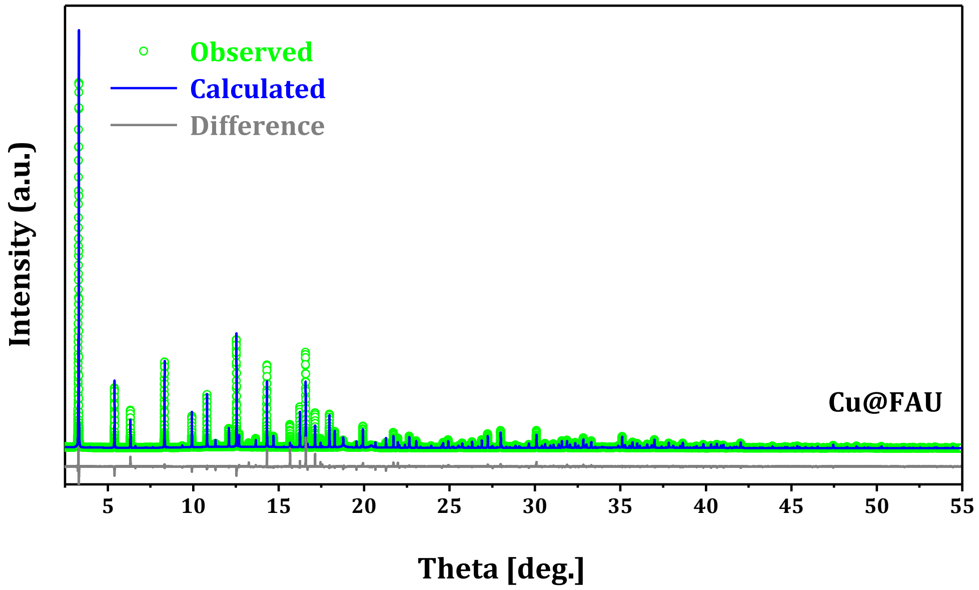

Figure S11 Rietveld refinements of synchrotron X-ray powder diffraction data of calcined Cu@FAU (λ=0.82452(1)Å, R_{wp}=11.5%; R_p=7.79%; Gof=2.95; R_{exp}=3.92%)

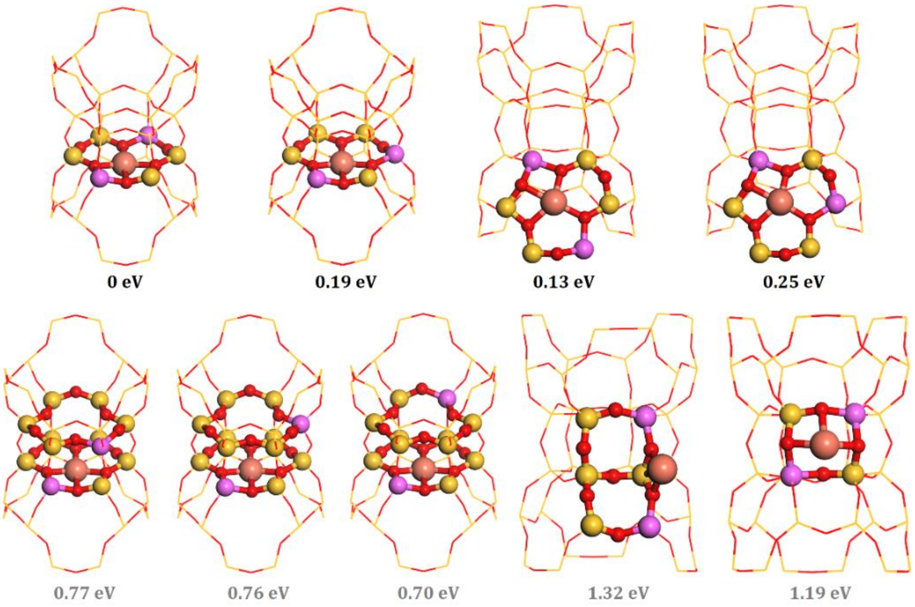

Figure S12 DFT-calculated structures of Cu@FAU with the relative energy shown in kJ/mol (Si: yellow; Al: pink; O: red; Cu: dark red). Lower energy indicates greater stability. The DFT study indicates that all structures shown in the upper level may exist in Cu@FAU

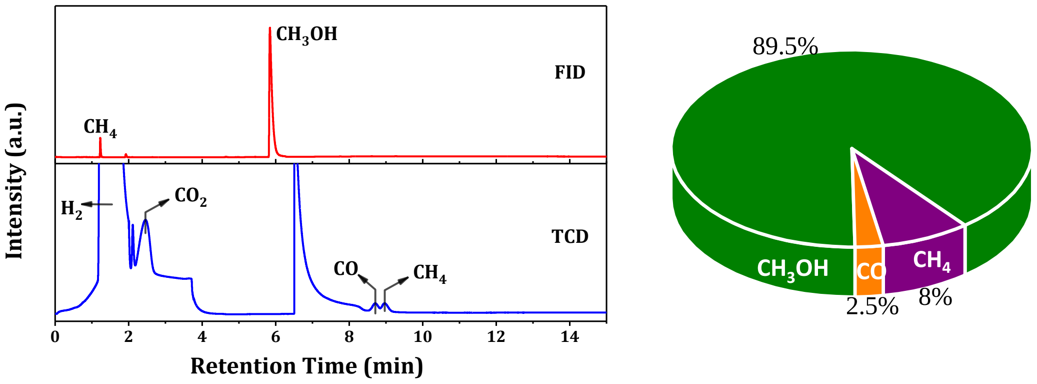

Figure S13 Product distribution from CO_2 hydrogenation over Cu@FAU catalyst. Reaction conditions: 0.15 g catalyst, H_2/CO_2 = 3/1, 513 K, GHSV= 12000 h^{-1}

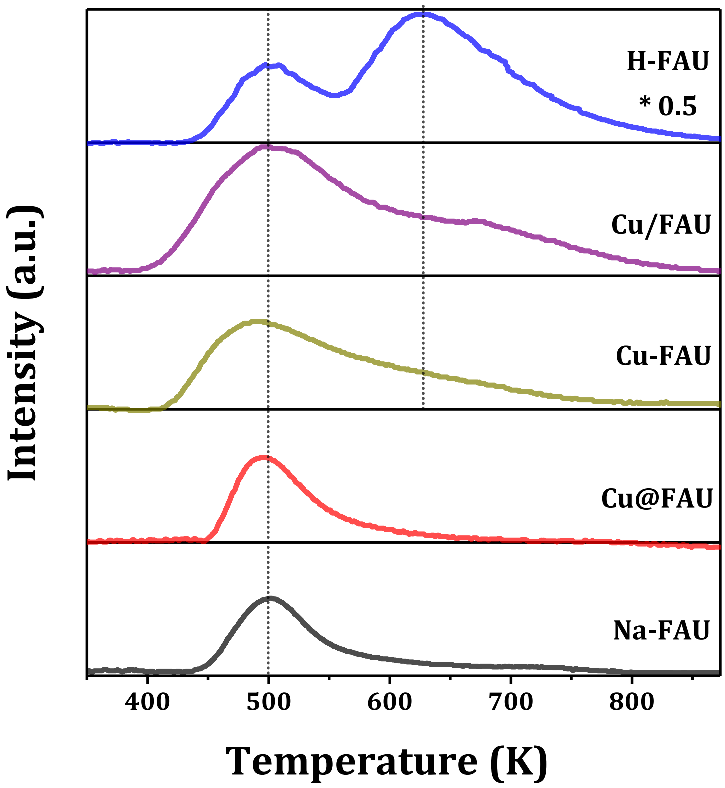

Figure S14 NH_3-TPD profiles of H-FAU, Na-FAU and Cu-containing FAU zeolites

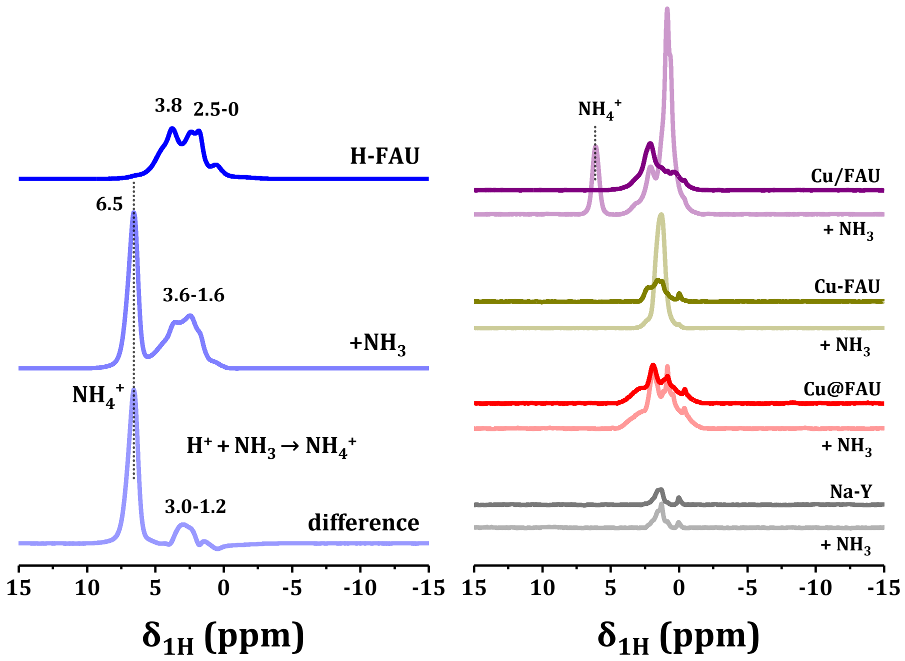

Figure S15 ^{1}H MAS NMR spectra of H-FAU, Na-FAU and Cu-containing FAU zeolites recorded before and after ammonia adsorption

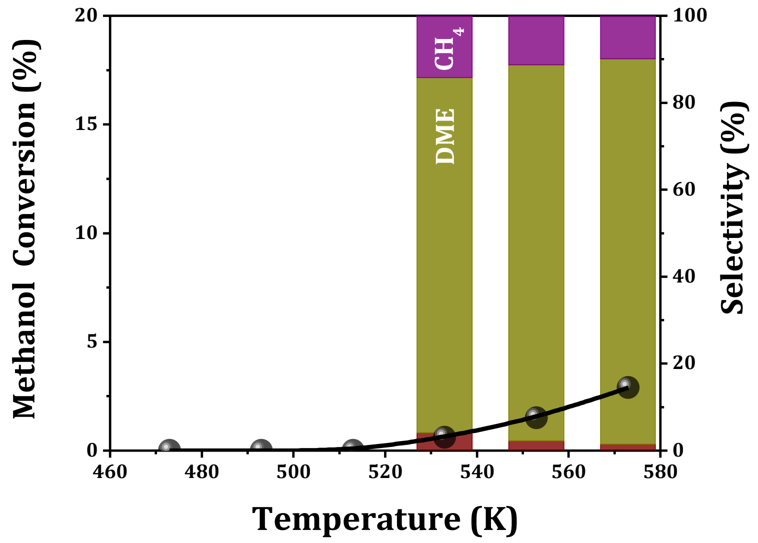

Figure S16 Methanol dehydration over Cu@FAU catalyst. Reaction conditions: 0.15 g catalyst, 3 MPa, 513 K, WHSV= 1.0 h^{-1}

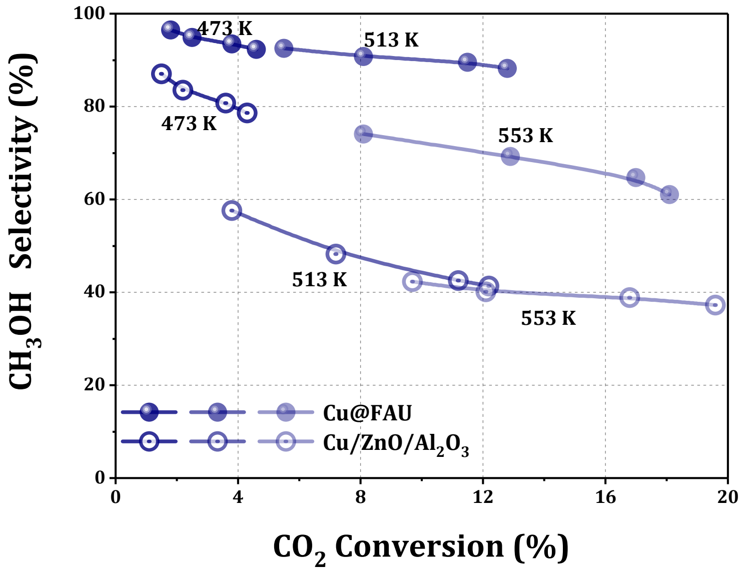

Figure S17 Comparison of CH_3OH selectivity in CO_2 hydrogenation over Cu@FAU and $Cu/ZnO/Al_2O_3$ catalysts. Reaction conditions: 0.15 g catalyst, H_2/CO = 3/1, 3 Mpa, 473-553 K, GHSV= 8000-20000 h^{-1}

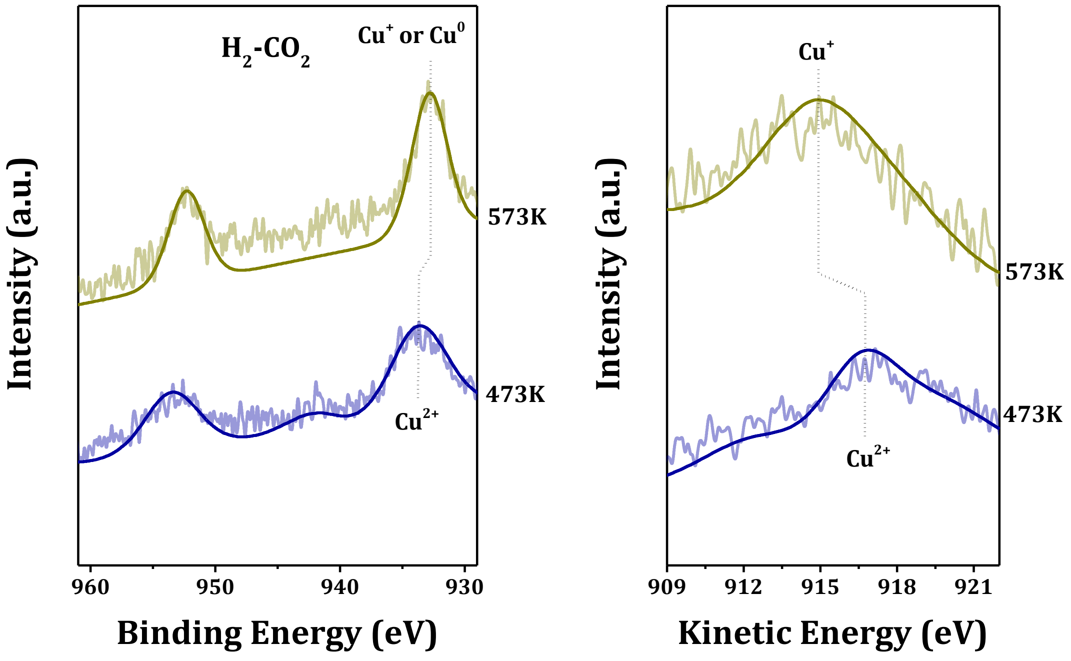

Figure S18 *In situ* near-ambient pressure X-ray photoelectron spectra of Cu@FAU during CO_2 hydrogenation

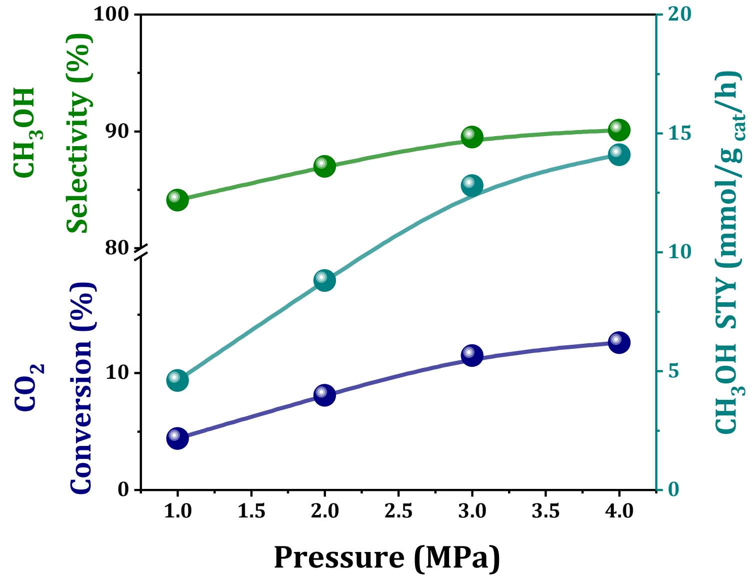

Figure S19 Effect of pressure on CO_2 hydrogenation over Cu@FAU catalyst. Reaction conditions: 0.15 g catalyst, H_2/CO_2 = 3/1, 513 K, GHSV= 12000 h^{-1}

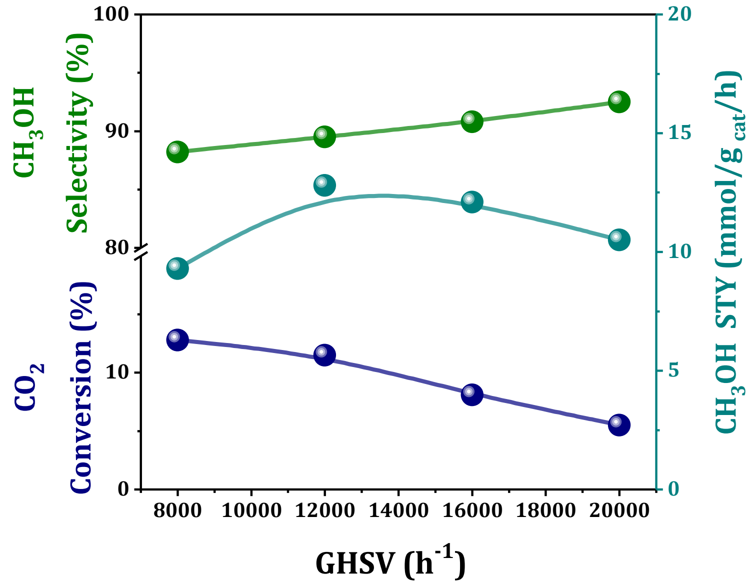

Figure S20 Effect of GHSV on CO_2 hydrogenation over Cu@FAU catalyst. Reaction conditions: 0.15 g catalyst, H_2/CO_2 = 3/1, 3 MPa, 513 K

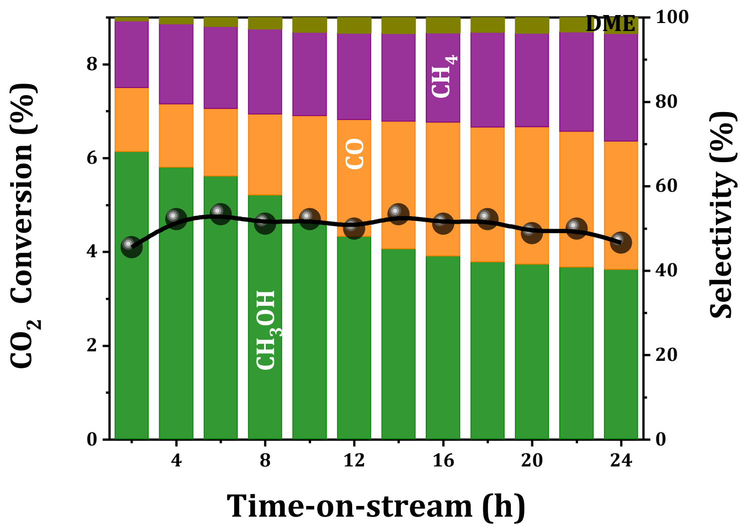

Figure S21 Time-on-stream behaviors of CO_2 hydrogenation over Cu-FAU catalyst. Reaction conditions: 0.15 g catalyst, H_2/CO_2 = 3/1, 513 K, GHSV= 12000 h^{-1}

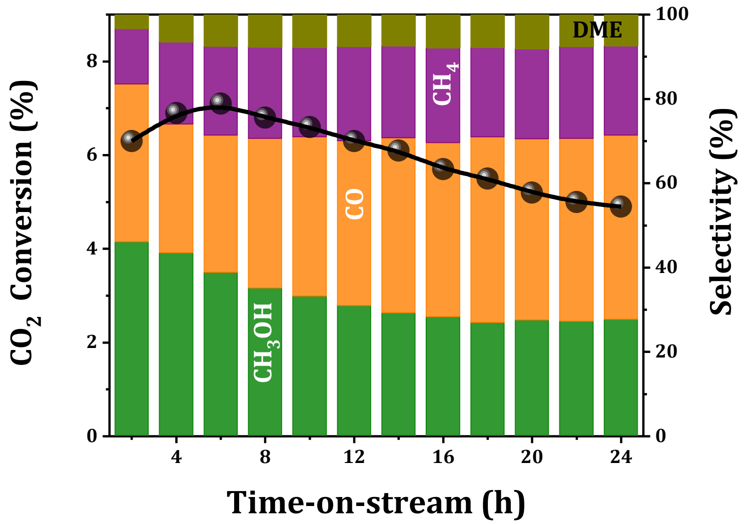

Figure S22 Time-on-stream behaviors of CO_2 hydrogenation over Cu/FAU catalyst. Reaction conditions: 0.15 g catalyst, H_2/CO_2 = 3/1, 513 K, GHSV= 12000 h^{-1}

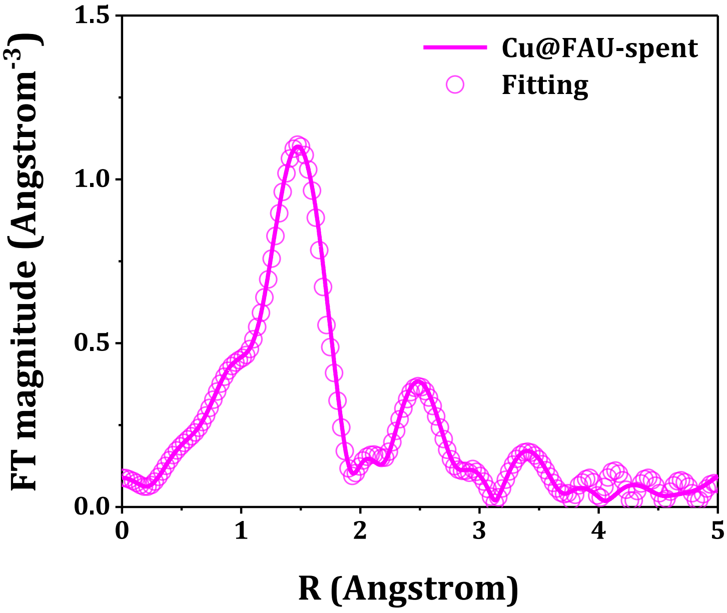

Figure S23 FT EXAFS fitting spectrum of Cu@FAU-spent

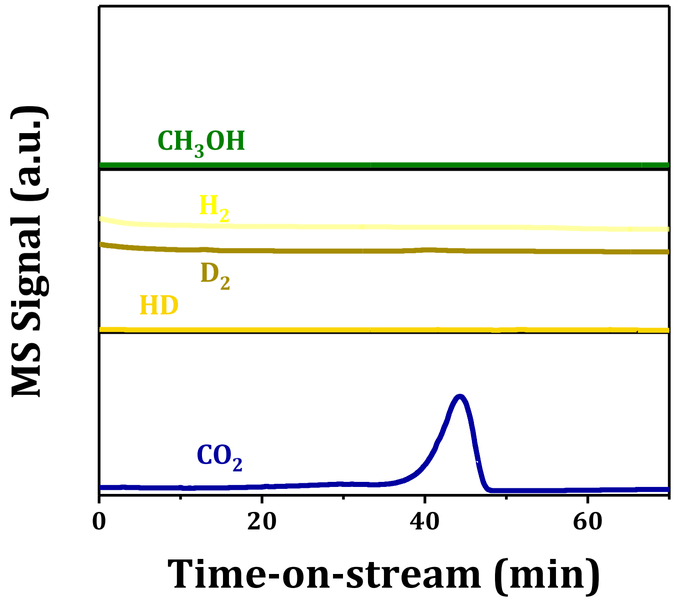

Figure S24 Mass spectrometry responses of CO_2 pulses fed to quartz sand in H_2-D_2 stream. Reaction conditions: 0.2 g catalyst, 0.4 MPa, 513 K, 5 mL/min CO_2, 15 mL/min H_2-D_2 (1/1)

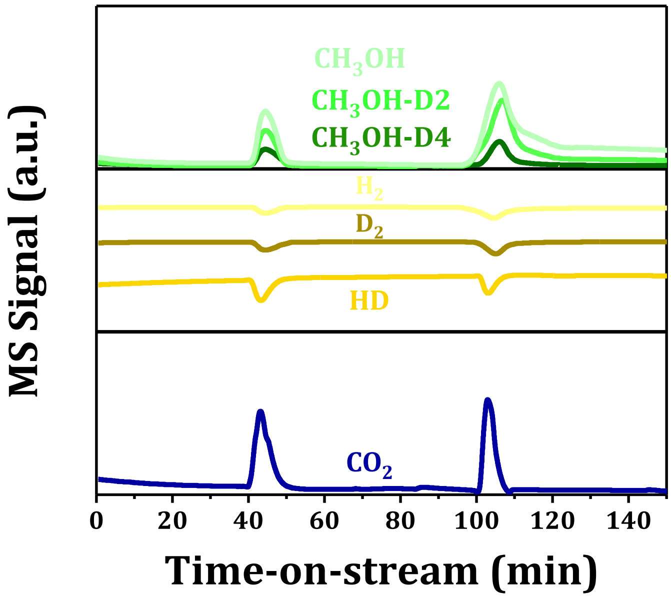

Figure S25 Mass spectrometry responses of CO_2 pulses fed to $Cu/ZnO/Al_2O_3$ in H_2-D_2 stream. Reaction conditions: 0.2 g catalyst, 0.4 MPa, 513 K, 5 mL/min CO_2, 15 mL/min H_2-D_2 (1/1)

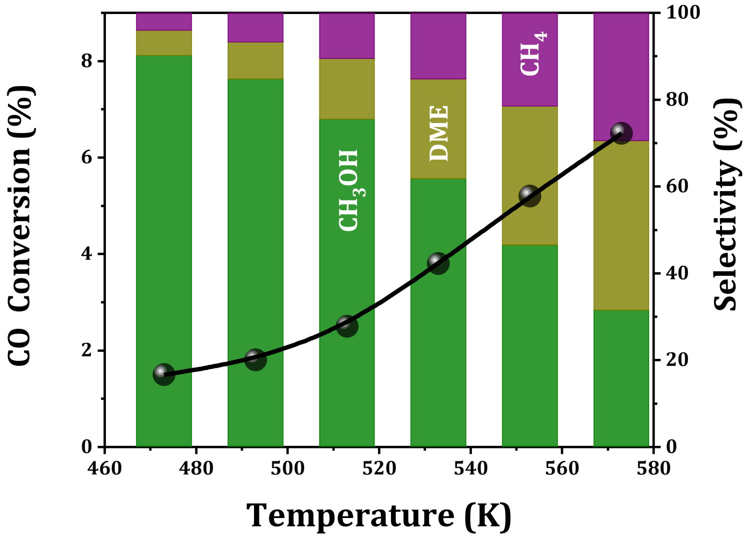

Figure S26 CO hydrogenation over Cu@FAU catalyst. Reaction conditions: 0.15 g catalyst, H_2/CO = 3/1, 3 Mpa, 513 K, GHSV= 12000 h^{-1}

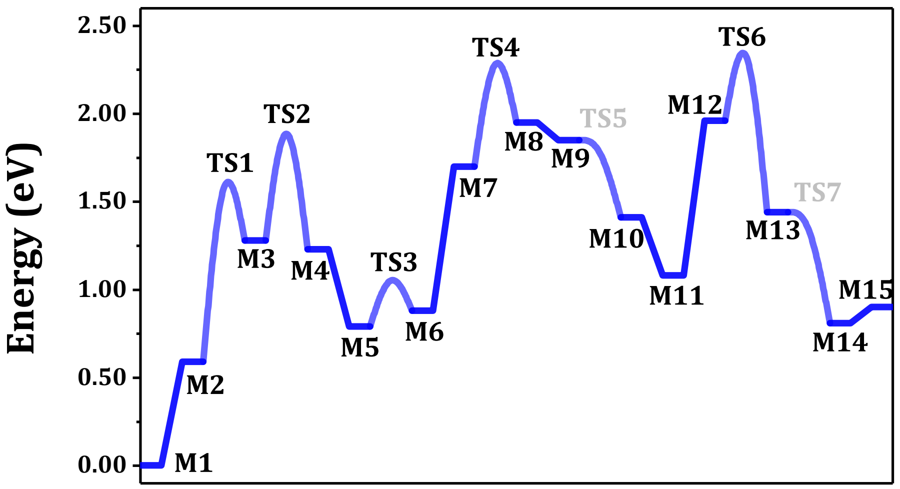

Figure S27 Energy profile of CO_2 hydrogenation to CH_3OH over Cu@FAU catalyst at 513 K

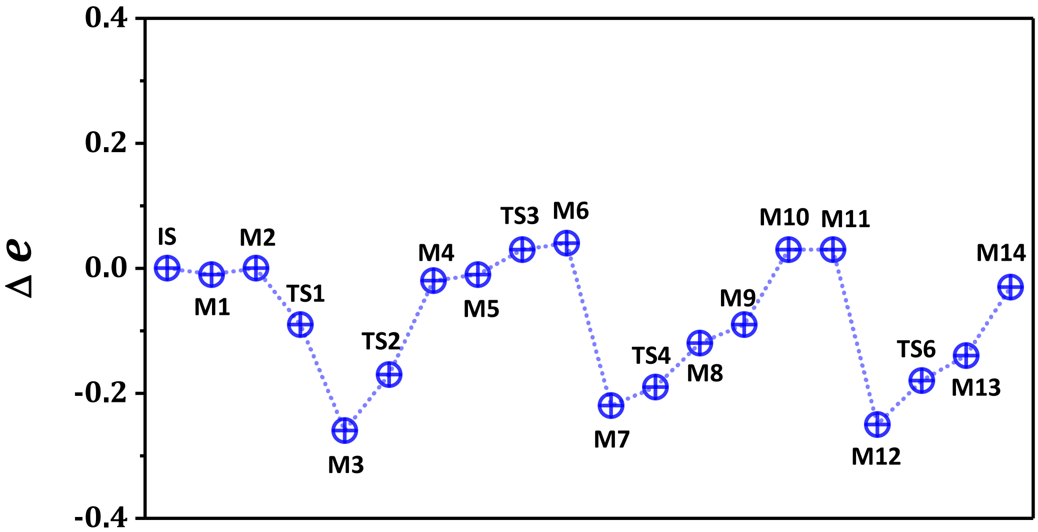

Figure S28 Bader charge analyses of copper during CO_2 catalytic reduction

Table S1 Physico-chemical properties of Cu containing zeolites under study

Sample	Si/Al [a]	Cu content (wt%) [a]	Surface area (m^2g^{-1}) [b]	Pore volume (cm^3g^{-1}) [c]
Cu@FAU before ion-exchanged with Na^+	3.2	4.7	533	0.37
Cu@FAU	3.2	4.5	538	0.37
Cu-FAU	3.5	5.2	612	0.39
Cu/FAU	3.5	4.9	606	0.38

[a]: Determined by ICP analyses; [b]: Determined by low-temperature Ar adsorption; [c]: Calculated from t-plot method

Table S2 EXAFS data fitting results of Cu-containing samples [a]

Sample	Shell	N [b]	R (Å) [c]	σ^2 x10^2 (Å^2) [d]	ΔE_0 (eV) [e]	r-factor (%)
Cu Foil	Cu-Cu	12.0	2.54	0.86	3.8	0.4
CuO	Cu-O	4.0	2.02	0.57	2.6	0.8
	Cu-Cu	4.0	2.96	0.82	5.3	/
Cu@FAU	Cu-O	3.8	2.02	0.65	-2.5	1.1
	Cu-Si/Al	2.0	2.77	0.95	8.9	/
Cu@FAU-spent	Cu-O	3.9	2.01	0.74	-2.8	0.9
	Cu-Si/Al	2.0	2.78	0.92	5.9	/

[a]: The data range used for fitting in k-space (Δk) and R-space (ΔR) are 3.0-12.2 Å^{-1} and 1.0-2.0 Å, respectively; [b]: Coordination number for the absorber-backscatterer pair; [c]: Average absorber–backscatterer distance; [d]: Debye-Waller factor; [e]: Inner potential correction. The accuracies of the above parameters were estimated as N, ±20%; R, ±1%; σ^2, ±20%; ΔE_0, ±20%.

Table S3 Methanol STY of the Cu@FAU compared to other Cu-based catalysts reported in the literature

Sample	Pressure (Mpa)	GHSV (h^{-1})	Temp. (K)	CO_2 Conv. (%)	CH_3OH Sel. (%)	STY_{CH3OH} (mmol/g_{cat}/h)	Ref.
Cu@FAU	3.0	12000	513	11.5	89.5	12.8	This work
	4.0	12000	513	12.6	90.1	14.1	
$Cu/ZnO/Al_2O_3$	3.0	12000	513	11.2	42.5	6.0	
	3.0	12000	553	16.8	38.8	8.2	
$Cu/ZnO/Al_2O_3$	5.0	15000	513	11.8	46.0	9.4	20
$Cu/ZnO/Al_2O_3$	5.0	12000	523	19.7	39.7	10.6	S10
$Cu/ZnO/Al_2O_3$	5.0	18000	523	11.1	54.8	11.5	11
$Cu/ZnO/Al_2O_3$	5.0	10000	503	13.4	58.1	7.8	S11
$Cu/ZnO/Al_2O_3$	6.0	30000	483	/	/	11.2	S12
$Cu/ZnO/Ga_xO$	4.5	18000	503	9.6	65.2	13.1	S13
Pd-Cu/ZnO	4.5	10800	513	5.8	73.0	5.0	S14
CuZn@UiO	4.0	18000	523	3.3	100	6.2	11
Cu-In-Zr-O	2.5	18000	523	1.5	79.8	2.5	S15
Cu/AlCeO	3.0	14400	513	10.1	64.2	10.3	S16
$CuNi/CeO_2$	3.0	14400	533	16.9	43.8	11.8	S17
	3.0	15000	513	5.6	77.6	5.9	
$Cu/La_2O_2CO_3$	3.0	12000	513	5.6	92.5	6.6	S18
$CuO-ZnO-ZrO_2$	5.0	28600	513	9.3	47.0	9.1	S19
Cu/SiO_2	5.0	28600	533	15.1	42	13.4	S20
	3.0	16000	533	8.1	40.3	4.4	

$Cu/CeO_x/TiO_2$	0.5	/	600	/	/	/	10
Cu/Al_2O_3	10.0	4000	473	2.4	46.2	1.1	S21
$Cu\text{-}K/Al_2O_3$	10.0	4000	473	2.8	62.2	1.6	S22
$Cu/ZnO/ZrO_2$	3.0	/	503	19.6	44.4	2.3	
$Cu/Zr_{0.9}@SiO_2$	2.5	16000	523	/	69.0	1.1	12
Cu/ZrO_2	2.5	20800	523	/	75.0	1.2	13
$Cu/Mo_2CT_x/SiO_2$	2.5	/	523	/	52.0	1.9	14
Cu/ZrO_2	/	39600	493	0.3	27.0	1.4	27
$Cu/Ti@SiO_2$	2.5	/	503	<10	85.0	3.0	S23
CuLa/SBA-15	2.5	/	513	5.7	81.2	6.0	S24
Cu/ZrO_2	1.0	/	503	4.2	39.0	1.2	S25
Cu/ZrO_2	8.0	3600	533	15.0	86.0	6.5	S26
CuZnZr/SBA-15	3.0	6600	523	19.2	30.6	11.7	S27
CuZn/NrGO	1.5	2444	523	24.2	/	12.6	S28
$Cu@m\text{-}SiO_2$	5.0	/	523	10.2	26.5	1.8	S29
$Cu/ZnO@m\text{-}SiO_2$	5.0	/	523	9.8	66.6	4.3	S29
$CuO/ZnO/ZrO_2$	3.0	/	523	5.3	83	2.0	S30
$Cu/ZnO/Al_2O_3/ZrO_2$	3.0	4000	523	25.9	49.2	7.3	S31
$CuO/ZnO/ZrO_2$	5.0	10000	553	22.2	34.0	10.8	S32
$CuO/ZnO/ZrO_2$	5.0	3000	523	11.4	92.7	3.3	S33
$CuO/ZnO/ZrO_2$	3.0	/	513	16.8	41.4	1.6	S34
$CuO/ZnO/TiO_2/ZrO_2$	3.0	/	513	16.2	43.7	1.7	S35
$Cu/ZnO/ZrO_2$	3.0	/	493	18.9	80.2	9.3	S36
$CuO/ZnO/Al_2O_3$	3.0	3600	513	14.6	63.6	3.8	S37

$CuO/ZnO/Al_2O_3$	4.0	/	513	59.5	73.4	4.1	S38
$Cu/ZnO/Al_2O_3/ZrO_2$	5.0	/	523	25.9	61.5	6.8	S39

Table S4 Stretching bands of key intermediates involved in CO_2 hydrogenation from DFT calculations

Species	Bands and vibration frequencies							
ln-CO_2*	ν_{as}(C-O)	ν_s(C-O)					–	–
	2373	1326						
mono-HCOO*	ν(C-H)	ν(C-O)+σ(C-O)	σ(C-H)	ν(C-O)	σ_w(H-C-O)			
	3009	1490	1317	1249	1000			
bi-HCOO*	ν(C-H)	ν_{as}(C-O)+σ(C-H)	ν_s(C-O)	ν_{as}(C-O)	σ_w(H-C-O)			
	3107	1464	1354	1259	1014			
HCOOH*	ν(O-H)	ν(C-H)	ν(C-O)	σ(C-H)+ν(C-O)	ν(C-O)+σ(C-O)	ν(C-O)+σ(O-H)	σ_w(H-C-O)	
	3646	3084	1685	1399	1331	1152	1038	
H_2COOH*	ν(O-H)	ν(C-H)	ν(C-H)	σ_s(C-H)	σ_w(C-H)	σ(C-O-H)	σ_t(C-H)	ν(C-O)+σ_w(C-H)
	3717	2962	2916	1390	1332	1318	1193	1040
H_2O*	ν(O-H)	ν(O-H)	σ_s(O-H)					
	3783	3234	1615					
[CH_2O-H_2O]*	ν_{as}(C-H)	ν_s(C-H)	ν(C-O)+σ_s(C-H)	ν(C-O)+σ_s(C-H)	σ_r(H-C-O)	σ_w(H-C-O)		
	3107	3033	1592	1463	1212	1158		
CH_3O*	ν(C-H)	ν_{as}(C-H)	ν_s(C-H)	σ_w (C-H)	σ_s(C-H)	σ_s(C-H)	σ(H-C-O)	σ(H-C-O)
	3027	2974	2936	1424	1395	1386	1091	1033
CH_3OH*	ν(O-H)	ν_{as}(C-H)	ν_{as}(C-H)	ν_s(C-H)	σ_s(C-H)	σ_w(C-H)	σ(H-C-O)	σ(H-C-O)
	3164	3127	3095	3061	1493	1464	1167	1150
CH_4*	ν_{as}(C-H)	ν_{as}(C-H)	ν_{as}(C-H)	ν_s(C-H)	σ_s(C-H)	σ_s(C-H)	σ_w (C-H)	
	3120	3111	3096	3044	1555	1554	1338	
CO (g)	ν(C-O)							
	2129							
CO_2 (g)	ν_{as}(C-O)	ν_s(C-O)						
	2376	1318						
CH_4 (g)	ν_{as}(C-H)	ν_{as} (C-H)	ν_s(C-H)	σ_s(C-H)	σ_s(C-H)	σ_w(C-H)	σ_w(C-H)	
	3121	3120	3007	1558	1555	1336	1331	
CH_3OH (g)	ν(O-H)	ν_{as}(C-H)	ν_{as}(C-H)	ν_s(C-H)	σ_s(C-H)	σ_w(C-H)+ν(C-O)	σ(C-O-H)	σ_r(C-H)
	3791	3093	3014	2942	1493.14	1460	1364	1156

ν: stretching vibrations; ν_{as}: asymmetric stretching vibrations; ν_s: symmetric stretching vibrations; σ: bending vibrations; σ_s: in-plane scissoring vibrations; σ_r: in-plane rocking vibrations; σ_w: out-of-plane wagging vibrations; σ_t: out-of-plane twisting vibrations; a+b means contribution from two types of vibrations, with dominant contribution shown in the front; Observed IR bands at 1250, 1335, 1385, 1465, 1495, 1585 and 2915 cm^{-1}, as highlighted in the table.

Table S5 All intermediates in the energy profile in **Figure 4**

	Intermediates
M1	*+CO_2(g)+3H_2(g)
M2	CO_2*+3H_2(g)
M3	[CO_2+H_{-Cu}+H_{-O}]*+2H_2(g)
M4	[mono-HCOO+H_{-O}]*+2H_2(g)
M5	[bi-HCOO+H_{-O}]*+2H_2(g)
M6	HCOOH*+2H_2(g)
M7	[HCOOH+H_{-Cu}+H_{-O}]*+H_2(g)
M8	[H_2COOH+H_{-O}]*$_{-1}$+H_2(g)
M9	[H_2COOH+H_{-O}]*$_{-2}$+H_2(g)
M10	[CH_2O+H_2O]*+H_2(g)
M11	CH_2O*+H_2O(g)+H_2(g)
M12	[CH_2O+H_{-Cu}+H_{-O}]*+H_2O(g)
M13	[CH_3O+H_{-O}]*+H_2O(g)
M14	CH_3OH*+H_2O(g)
M15	*+CH_3OH(g)+H_2O(g)

Table S6 Adsorption energy of key intermediates involved in CO_2 hydrogenation

Adsorbates	E_{ads} (eV)	
	at 0 K	at 513 K
CO_2*	-0.22	0.59
mono-HCOO*	-0.60	0.03
bi-HCOO*	-0.94	-0.18
HCOOH*	-0.75	-0.11
H_2COOH*	-0.98	-0.09
H_2O*	-0.58	0.09
CH_2O*	-0.61	-0.01
CH_3O*	-0.97	-0.26
CH_3OH*	-0.85	-0.09

Supplementary References

[S1] Yu H, Wei X and Li J *et al.* The XAFS beamline of SSRF. *Nucl Sci Tech* 2015; **26:** 050102.

[S2] Kresse G and Furthmülle J. Efficiency of ab-initio total energy calculations for metals and semiconductors using a plane-wave basis set. *Comp Mater Sci* 1996; **6:** 15-50.

[S3] Kresse G and Furthmülle J. Efficient iterative schemes for ab initio total-energy calculations using a plane-wave basis set. *Phys Rev B* 1996; **54:** 11169-11186.

[S4]Perdew JP, Burke K and Ernzerhof M. Generalized gradient approximation made simple. *Phys Rev Lett* 1996; **77:** 3865-3868.

[S5] Blöchl PE. Projector augmented-wave method. *Phys Rev B* 1994; **50:** 17953-17979.

[S6] Wellendorff J, Lundgaard K and Møgelhøj A. Density functionals for surface science: Exchange-correlation model development with Bayesian error estimation. *Phys Rev B* 2012; **85:** 235149.

[S7] Sun L, Wang Y and Wang C. Water-involved methane-selective catalytic oxidation by dioxygen over copper zeolites. *Chem* 2021; **7:** 1557-1568.

[S8] Henkelman G and Jónsson H. Improved tangent estimate in the nudged elastic band method for finding minimum energy paths and saddle points. *J Chem Phys* 2000; **113:** 9978-9985.

[S9] Henkelman G, Uberuaga B and Jónsson H. A climbing image nudged elastic band method for finding saddle points and minimum energy paths. *J Chem Phys* 2000; **113:** 9901-9904.

[S10] Gao P, Li F and Zhao N et al. Influence of modifier (Mn, La, Ce, Zr and Y) on the performance of Cu/Zn/Al catalysts via hydrotalcite-like precursors for CO_2 hydrogenation to methanol. *Appl Catal A* 2013; **468:** 442-452.

[S11] Gao P, Zhong L and Zhang L et al. Yttrium oxide modified Cu/ZnO/Al_2O_3 catalysts via hydrotalcite-like precursors for CO_2 hydrogenation to methanol. *Catal Sci Technol 2015;* **5:** 4365-4377.

[S12] Ruland H, Song H and Laudenschleger D et al. CO_2 hydrogenation with Cu/ZnO/Al_2O_3: A benchmark study. *ChemCatChem* 2020; **12:** 3216-3222.

[S13] Li M, Chen C and Suo H et al. CO_2 hydrogenation to methanol over catalysts derived from single cationic layer CuZnGa LDH precursors. *ACS Catal* 2018; **8:** 4390-4401.

[S14] Hu B, Yin Y and Liu G et al. Hydrogen spillover enabled active Cu sites for methanol synthesis from CO_2 hydrogenation over Pd doped CuZn catalysts. *J Catal* 2018; **389:** 17-26.

[S15] Yao L, Shen X and Pan Y *et al.* Z. Synergy between active sites of Cu-In-Zr-O catalyst in CO_2 hydrogenation to methanol. *J. Catal* 2019; **372:** 74-85.

[S16] Li S, Guo L and Ishihara T *et al*. Hydrogenation of CO2 to methanol over Cu/AlCeO catalyst. *Catal Today* 2020; **339:** 352-361.

[S17] Tan Q, Shi Z and Wu D *et al.* CO_2 hydrogenation to methanol over a highly active Cu-Ni/CeO_2-nanotube catalyst. *Ind Eng Chem Res* 2018; **57:** 10148-10158.

[S18] Chen K, Duan X and Fang H et al. Selective hydrogenation of CO_2 to methanol catalyzed by Cu supported on rod-like $La_2O_2CO_3$. *Catal Sci Technol* 2018; **8:** 1062-1069.

[S19] Angelo L, Girleanu M and Ersen O et al. Catalyst synthesis by continuous coprecipitation under micro-fluidic conditions: Application to the preparation of catalysts for methanol synthesis from CO_2/H_2. *Catal Today* 2016; **270:** 59-67.

[S20] Wang Z, Xu Z and Peng S et al. High-performance and long-lived Cu/SiO_2 nanocatalyst for CO_2 hydrogenation. *ACS Catal.* 2015; **5:** 4255-4259 (2015).

[S21] Bansode A, Tidona B and von Rohr P. Urakawa, A. Impact of K and Ba Promoters on CO_2 Hydrogenation over Cu/Al_2O_3 Catalysts at High Pressure. *Catal Sci Technol* 2013; **3:** 767−778.

[S22] Ban H, Li C and Asami K *et al.* Influence of Rare-Earth Elements (La, Ce, Nd and Pr) on the Performance of Cu/Zn/Zr Catalyst for CH_3OH Synthesis from CO_2. *Catal Commun* 2014; **54:** 50-54.

[S23] Noh G, Lam E and Alfke J *et al.* Selective Hydrogenation of CO_2 to CH_3OH on Supported Cu Nanoparticles Promoted by Isolated Ti(IV) Surface Sites on SiO_2. *ChemSusChem* 2019; **12:** 968-972.

[S24] Chen K, Fang H and Wu S *et al.* CO_2 Hydrogenation to Methanol over Cu Catalysts Supported on La-Modified SBA-15: The Crucial Role of Cu-LaOx Interfaces. *Appl Catal B* 2019; **251:** 119-129.

[S25] Tada S and Satokawa S. Effect of Ag Loading on CO_2-to-Methanol Hydrogenation over Ag/CuO/ZrO_2. *Catal Commun* 2018; **113:** 41-45.

[S26] Samson K, Śliwa M and Socha R *et al.* Influence of ZrO_2 Structure and Copper Electronic State on Activity of Cu/ZrO_2 Catalysts in Methanol Synthesis from CO_2. *ACS Catal* 2014; **4:** 3730-3741.

[S27] Mureddu M, Ferrara F and Pettinau A. Highly Efficient CuO/ZnO/ZrO_2@SBA-15

Nanocatalysts for Methanol Synthesis from the Catalytic Hydrogenation of CO_2. *Appl Catal B* 2019; **258:** 117941-117954.

[S28] Deerattrakul V, Yigit N and Rupprechter G *et al.* The Roles of Nitrogen Species on Graphene Aerogel Supported Cu-Zn as Efficient Catalysts for CO_2 Hydrogenation to Methanol. *Appl Catal A* 2019; **580:** 46–52.

[S29] Yang H, Gao P and Zhang C *et al.* Core-Shell Structured Cu@m-SiO_2 and Cu/ZnO@m-SiO_2 Catalysts for Methanol Synthesis from CO_2 Hydrogenation. *Catal Commun* 2016; **84:** 56-60.

[S30] Fang X, Men Y and Wu F *et al.* Moderate-Pressure Conversion of H_2 and CO_2 to Methanol via Adsorption Enhanced Hydrogenation. *Int J Hydrogen Energy* 2019; **44:** 21913-21925.

[S31] Hou X, Xu C and Liu Y *et al.* Improved Methanol Synthesis from CO_2 Hydrogenation over CuZnAlZr Catalysts with Precursor pre-Activation by Formaldehyde. *J Catal* 2019; **379:** 147-153.

[S32] Angelo L, Girleanu M and Ersen O *et al.* Catalyst Synthesis by Continuous Coprecipitation under Micro-Fluidic Conditions: Application to the Preparation of Catalysts for Methanol Synthesis from CO_2/H_2. *Catal Today* 2016; **270:** 59-67.

[S33] Chen S, Zhang J and Wang P *et al.* Effect of Vapor-Phase-Treatment to CuZnZr Catalyst on the Reaction Behaviors in CO_2 Hydrogenation into Methanol. *ChemCatChem* 2019; **11:** 1448-1457.

[S34] Chen D, Mao D and Wang G *et al.* CO_2 Hydrogenation to Methanol over CuO-ZnO-ZrO_2 Catalyst Prepared by Polymeric Precursor Method. *J Sol-Gel Sci Technol* 2019; **89:** 686-699.

[S35] Chen D, Mao D and Xiao J *et al.* CO_2 Hydrogenation to Methanol over CuO-ZnO-TiO_2-ZrO_2: A Comparison of Catalysts Prepared by Sol-Gel, Solid-State Reaction and SolutionCombustion. *J Sol-Gel Sci Technol* 2018; **86:** 719-730.

[S36] Wang Y, Kattel S and Gao W *et al.* Exploring the Ternary Interactions in Cu-ZnO-ZrO_2 Catalysts for Efficient CO_2 Hydrogenation to Methanol. *Nat Commun* 2019; **10:** 1166-1176.

[S37] Lei H, Hou Z and Xie J. Hydrogenation of CO_2 to CH_3OH over CuO/ZnO/Al_2O_3 Catalysts Prepared via A Solvent-Free Routine. *Fuel* 2016; **164:** 191-198.

[S38] Wu W, Xie K and Sun D *et al.* Cu/ZnO/Al_2O_3 Catalyst Prepared by Mechanical-Force-Driven Solid-State Ion Exchange and Its Excellent Catalytic Activity under Internal Cooling Condition. *Ind Eng Chem Res* 2017; **56:** 8216-8223.

[S39] Zhang Y, Zhong L and Wang H *et al.* Catalytic Performance of Spray-Dried Cu/ZnO/Al_2O_3/ZrO_2 Catalysts for Slurry Methanol Synthesis from CO_2 Hydrogenation. *J CO_2 Util*

2016; **15:** 72-82.
